# Closed genomes of commercial inoculant rhizobia provide a blueprint for management of legume inoculation

**DOI:** 10.1128/aem.02213-24

**Published:** 2025-01-10

**Authors:** MacLean G. Kohlmeier, Graham W. O'Hara, Joshua P. Ramsay, Jason J. Terpolilli

**Affiliations:** 1 Legume Rhizobium Sciences, Food Futures Institute, Murdoch University 685868 , Murdoch, Western Australia, Australia; 2 Curtin Medical School, Curtin University 614117 , Bentley, Western Australia, Australia; 3 Curtin Health Innovation Research Institute, Curtin University 1649 , Bentley, Western Australia, Australia; University of Tennessee at Knoxville, Knoxville, Tennessee, USA

**Keywords:** genomics, horizontal gene transfer, inoculants, legume, symbiosis, DNA sequencing

## Abstract

Rhizobia are soil bacteria capable of establishing symbiosis within legume root nodules, where they reduce atmospheric N_2_ into ammonia and supply it to the plant for growth. Australian soils often lack rhizobia compatible with introduced agricultural legumes, so inoculation with exotic strains has become a common practice for over 50 years. While extensive research has assessed the N_2_-fixing capabilities of these inoculants, their genomics, taxonomy, and core and accessory gene phylogeny are poorly characterized. Furthermore, in some cases, inoculant strains have been developed from isolations made in Australia. It is unknown whether these strains represent naturalized exotic organisms, native rhizobia with a capacity to nodulate introduced legumes, or recombinant strains arising from horizontal transfer between introduced and native bacteria. Here, we describe the complete, closed genome sequences of 42 Australian commercial rhizobia. These strains span the genera, *Bradyrhizobium*, *Mesorhizobium*, *Methylobacterium*, *Rhizobium*, and *Sinorhizobium*, and only 23 strains were identified to species level. Within inoculant strain genomes, replicon structure and location of symbiosis genes were consistent with those of model strains for each genus, except for *Rhizobium* sp. SRDI969, where the symbiosis genes are chromosomally encoded. Genomic analysis of the strains isolated from Australia showed they were related to exotic strains, suggesting that they may have colonized Australian soils following undocumented introductions. These genome sequences provide the basis for accurate strain identification to manage inoculation and identify the prevalence and impact of horizontal gene transfer (HGT) on legume productivity.

**IMPORTANCE:**

Inoculation of cultivated legumes with exotic rhizobia is integral to Australian agriculture in soils lacking compatible rhizobia. The Australian inoculant program supplies phenotypically characterized high-performing strains for farmers but in most cases, little is known about the genomes of these rhizobia. Horizontal gene transfer (HGT) of symbiosis genes from inoculant strains to native non-symbiotic rhizobia frequently occurs in Australian soils and can impact the long-term stability and efficacy of legume inoculation. Here, we present the analysis of reference-quality genomes for 42 Australian commercial rhizobial inoculants. We verify and classify the genetics, genome architecture, and taxonomy of these organisms. Importantly, these genome sequences will facilitate the accurate strain identification and monitoring of inoculants in soils and plant nodules, as well as enable detection of horizontal gene transfer to native rhizobia, thus ensuring the efficacy and integrity of Australia’s legume inoculation program.

## INTRODUCTION

Legumes have long been recognized for their ability to improve soil fertility, which results from the N_2_-fixing symbiotic associations they can establish with root nodule-forming bacteria known as rhizobia (1). In Australia, agricultural forage and grain legumes are exotic species introduced following European colonization during the late 18th century (2). Australian soils lacked effective rhizobia compatible with these introduced legumes, which led to the practice of inoculation (3). Since the mid-1950s, this approach has been developed into an organized system, whereby strains are collected from edaphically matched regions of the world and screened for effective N_2_ fixation with target legumes in glasshouse experiments (4). Highly effective strains are further tested in field experiments, designed to assess their symbiotic capacity and saprophytic competence across hosts and a range of agroecological conditions (5). For ease of manufacture, commercial viability, and quality control, a single strain is selected for a compatible group of legumes, known as an inoculant group. Inoculant Mother Cultures are maintained within the Australian Inoculant Research Group (AIRG) and supplied to manufacturers.

The commercially available rhizobial inoculant strains cover almost 100 cultivated legume species (6) and are drawn from five of the 20 (7) currently recognized genera: *Bradyrhizobium*, *Mesorhizobium*, *Methylobacterium*, *Rhizobium*, and *Sinorhizobium* (also known as *Ensifer*) (8). Beyond this broad delineation, our understanding of the species diversity of inoculant strains is poor, which limits our capacity to identify these organisms in the field and accurately attribute N_2_ fixation and yield benefit from inoculation.

Horizontal gene transfer (HGT) complicates the management of legume inoculation. Transfer of symbiosis genes from inoculant strains to non-symbiotic rhizobia present in Australian soils can lead to the evolution of new legume symbionts that differ in their N_2_ fixation capacity. Rhizobial symbiosis genes (e.g., *nod*, *nif*, and *fix*) are generally considered to form part of the accessory genome, and as such are often found encoded chromosomally on symbiosis islands (SIs) or on plasmids (1). For instance, horizontal transfer of the symbiosis Integrative and Conjugative Element (ICE) from the *Cicer arietinum* inoculant strain *Mesorhizobium ciceri* CC1192 and *Biserrula pelecinus* strain *M. ciceri* WSM1271 to resident soil bacteria resulted in the evolution of new legume-nodulating rhizobia (9–13). In some cases, the evolved strains are effective symbionts (9, 10). However, poorly effective novel strains have also been observed, with a potential to out-compete an inoculant strain for nodulation of the target legume (10–13). Transfer of symbiosis plasmids (pSyms) has also been shown *in vitro* for several well-characterized *Rhizobium* and *Sinorhizobium* strains (14, 15), and while studies of sympatric populations suggest pSym transfer occurs frequently in the rhizosphere (16–19), the potential impacts of pSym transfer on symbiotic N_2_ fixation in the field have not been investigated.

The advent of whole genome sequencing (WGS) facilitated by NextSeq 500 and MinION Mk1B platforms has made the generation of complete genomes for rhizobia, which are generally between ~7 and 9 Mb in size, a cost-effective undertaking for a research laboratory. Complete bacterial genomes consist of a single high-quality and gap-free sequence for each bacterial DNA molecule (i.e., chromosome or plasmid(s)), providing an accurate replicon structure and an unbiased template for phylogenetic and evolutionary analyses (20). An array of genome-based taxonomy tools, such as digital DNA–DNA hybridization (dDDH), average nucleotide identity (ANI), and average amino acid identity (AAI), and the capacity to generate core genome phylogenetic analyses with many hundreds (21) or even thousands of shared single-copy genes (22) provide a high-resolution means of characterizing and classifying bacteria than was previously achievable. Furthermore, complete genome sequences allow for symbiosis gene loci to be accurately identified and their transfer in the environment following inoculation to be monitored (9, 10).

While most of the commercial inoculants used in Australia originated from other countries, 13 of the current inoculant strains were isolated from Australian soils. The origin of these strains has been debated (3, 23) with three possibilities being that they are (i) exotic, originating from previous undocumented deliberate or accidental introductions, and have subsequently colonized soils and become “naturalized strains”; (ii) native strains, which not only nodulated indigenous legumes prior to colonization but also have a capacity to also engage in symbiosis with introduced agricultural legumes; or (iii) recombinant strains, evolving through HGT between strains from (i) and (ii), generating new rhizobia capable of nodulating introduced legumes.

Significant gaps in our understanding of the taxonomy, genomic structure, and origin of many strains within the suite of commercial inoculants hamper current efforts to accurately quantify their efficacy. Here, we sought to address this dearth in knowledge by completely sequencing the genomes of all the legume inoculants commercially available in Australia. We provide an updated taxonomy of these bacteria, identify their replicon structures, and examine their relationship to each other, as well as to the broader set of publicly available genome sequences. We also analyze and speculate on the origins of inoculant strains originally isolated from Australia.

## MATERIALS AND METHODS

### Bacterial strains and culture conditions

Strains used in this study are listed in Table 1. Commercial inoculant strains (8) were sourced from the AIRG (at the Department of Primary Industries, New South Wales [Menangle, Australia]) collection of 2019 Mother Cultures, to

**TABLE 1:** Australian commercial rhizobial inoculants analyzed in this study

Strain (inoculant group)	Origin	Current recommended legume host(s)	First use^*a*^
5G1B	Australia	*Vigna angularis*	1999
CB82	Australia	*Stylosanthes guianensis* var. *guianensis, S. guianensis* var. *intermedia*, *S. humilis*, *S. viscosa*	1978
CB376 (L)	Africa	*Lotononis bainesii*	1963
CB627	DRC	*Desmodium intortum*, *D. uncinatum*	1958
CB756 (M)	Zimbabwe	*Clitoria ternatea*, *Calopogonium mucunoides*, *Macroptilium atropurpureum*, *M. lathyroides*, *Mucuna deeringiana*, *Neontonia wightii*, *Pueraria phaseoloides*	1962
CB782^*b*^	Kenya	*Trifolium semipilosum*	1969
CB1015 (I)	India	*Vigna mungo*, *V. radiata*, *V. unguiculata*	1977
CB1024 (J)	India	*Cajanus cajan*, *Lablab pupureus*, *Macrotyloma axillare*, *M. uniflorum*	1972
CB1650	Brazil	*Stylosanthes hamata*	1984
CB1717	Brazil	*Macroptilium bracteatum*	1999
CB1809 (H)	USA	*Glycine max*	1966
CB1923	Brazil	*Centrosema pascuorum*, *C. pubescens*	1972
CB2312	Australia	*Aeschynomene americana*, *A. falcata*	1975
CB3035	Australia	*Cyamopsis tetragonoloba*	1990
CB3060	Australia	*Leucaena leucocephala*	1987
CB3090	Sri Lanka	*Gliricidia* spp.	1989
CB3126	Mexico	*Desmanthus virgatus*	1993
CB3171	Nicaragua	*Calliandra* spp.	2001
CB3481	Brazil	*Stylosanthes seabrana*	1996
CC283b	Russia	*Trifolium ambiguum*	1995
CC511	USA	*Phaseolus coccineus*, *P. lunatus*, *P. vulgaris*	1953
CC829 (D)	USA	*Lotus pedunculatus*	1958
CC1192^*b*^ (N)	Israel	*Cicer arietinum*	1977
CC1502	Australia	*Chamaecytisus palmensis*	1993
CIAT3101	Colombia	*Arachis pintoi*	1998
NC92 (*P*)	Bolivia	*Arachis hypogaea*	1989
RRI128 (AL)	Australia	*Medicago albus*, *M. littoralis*, *M. sativa*, *M. tornata*	2000
SRDI969 (F)	Australia	*Vicia faba*	2024
SU277	Australia	*Trigonella foenumgraecum*	1953
SU303	Australia	*Lathyrus cicera*, *L. sativus*, *Vicia narbonensis*	1992
SU343^*b*^	USA	*Lotus corniculatus*	1961
TA1 (B)	Australia	*Trifolium alexandrium*, *T. dubium*, *T. fragiferum*, *T. glomeratum*, *T. hybridum*, *T. pratense*, *T. repens*	1956
WSM471	Australia	*Ornithopus compressus*, *O. perpusillus*, *O. pinnatus*, *O. sativus*, *O. compressus* X *sativus, Lupinus albus*, *L. angustifolius*, *L. cosentinii*, *L. luteus*	1996
WSM1115 (AM)	Greece	*Medicago murex*, *M. polymorpha*, *M. scutellata*, *M. sphaerocarpus*, *M. truncatula*, *M. rugrosa*	2002
WSM1274	Greece	*Vicia faba*	1998–2023
WSM1325^*b*^ (C)	Greece	*Trifolium clypeatum, T. glanuliferum, T. hirtum, T. incarnatum, T. michelianum, T. purpureum, T. resupinatum, T. spumosum, T. subterraneum, T. vesiculosum*	2004
WSM1455	Greece	*Vicia faba*	2002–2023
WSM1497^*b*^	Greece	*Biserrula pelecinus*	1999
WSM1558	Italy	*Biserrula pelecinus*	1997–1998
WSM1592	Italy	*Hedysarum coronarium*	2006
WSM4643 (E)	Italy	*Lens culinaris*, *Pisum sativum*, *Viciae daisycarpa*, *V. ervilia*, *V. sativa*	2024
WU425 (G)	Australia	*Lupinus albus*, *L. angustifolius*, *L. cosentinii*, *L. luteus, Ornithopus compressus*, *O. perpusillus*, *O. pinnatus*, *O. sativus*, *O. compressus* X *sativus*	1970

^*a*^ Reference for first use based on Bullard et al. (3) for all inoculants, except for SRDI969 (6), WSM1325 (24), WSM1592 (25), and WSM4643 (6). DRC, Democratic Republic of the Congo; USA, United States of America.

^*b*^ Strains sequenced prior to initiation of this project include *M. ciceri* CC1192 (26), *M. ciceri* WSM1497 (27), *M. jarvisii* SU343 (28), *R. hidalgonense* CB782 (29), and *Rhizobium* sp. WSM1325 (24).

ensure that sequenced accessions genotypically matched inoculant strains provided by inoculant manufacturers. Historical inoculants *M. ciceri* WSM1274 and *M. opportunistum* WSM1558 (3) were supplied from the International Legume Inoculant Genebank (ILIG) at Murdoch University (Perth, Australia). Rhizobia of the genera *Mesorhizobium*, *Rhizobium*, and *Sinorhizobium* were routinely cultured in Typtone-Yeast (TY) medium (30), on a gyratory shaker at 250 rpm, at 28°C for 1–2 days. *Bradyrhizobium* strains were grown in ½ lupin agar (½ LA) medium (31) or yeast extract mannitol (YEM) medium (32), on a gyratory shaker at 250 rpm, at 28°C for 2–3 days. *Methylobacterium* sp. CB376 was grown in ½ LA supplemented with 20 mM succinate.

### DNA manipulations

Genomic DNA for long-read MinION sequencing was isolated via phenol-chloroform extraction as previously described (33). DNA for short-read sequencing was isolated using DNeasy Blood and Tissue kit (Qiagen, Germany) as per the manufacturer’s instructions. DNA quality and quantity were assessed with a Nanodrop One spectrophotometer (Thermo Fisher Scientific) and gel electrophoresis using standard techniques (34).

### Genome sequencing, assembly, polishing, and annotation

Isolated genomes were sequenced using the MinION Mk1B platform (Oxford Nanopore Technologies, UK) to generate long-read sequence data, and the Illumina NextSeq 500 platform (Illumina, USA) to generate 150 bp paired-end sequence data. MinION data were basecalled using guppy v 4.2.2+effbaf8 (35). Reads were filtered based on quality and size using Nanofilt (36). Basecalled reads were assembled using flye v 2.8.3-b1695 generating circular contigs (37). Assemblies were polished with unfiltered nanopore reads using Racon v 1.4.17 (38) and Illumina reads using Pilon v 1.23 (39). Genome annotations were carried out using the NCBI Prokaryotic Genome Annotation Pipeline (PGAP) v 6.2 (40, 41). Polished assemblies were oriented such that genes responsible for chromosomal and plasmid replication, *dnaA* and *repA*, respectively, were set as start points for the contigs using Geneious Prime 2024.0.7 (https://www.geneious.com).

### Taxonomic assignment

Complete genome sequences were submitted to the Type Strain Genome Server (TYGS) (https://tygs.dsmz.de/) for taxonomic assignments. The server compares query sequences to a database of known type strains and reports three dDDH values, d_0_, d_4_, and d_6_, determined from different equations, with d_4_ often preferred as it incorporates sequence identity within homologous segments and minimizes the impact of using incomplete genomes (21, 42). A dDDH value ≥70 indicates that the query and subject sequences belong to the same species. Two additional genome-based similarity indices, ANI and AAI, were used to validate the findings from the TYGS. For these metrics, values ≥96% were considered to belong to the same species. ANI and AAI values were determined using fastANI v 1.32 (43) and EzAAI v 1.2.3 (44), respectively.

A complete sequence for strain TA1 (45) was deposited following the sequencing of TA1 accession sourced from the 2019 AIRG mother culture in our study. While the two sequences are near identical (ANI value of 99.998%), we opted to use the TA1 AIRG accession sequenced during our analysis, for consistent comparison with other strains sequenced in this cohort. For all four *Sinorhizobium* strains (*S. medicae* SU277, *S. medicae* WSM1115, *S. meliloti* RRI128, and *S. terangae* CB3126), the top TYGS hit was actually *Ensifer* spp. In 2002, *Sinorhizobium* spp. were assigned to *Ensifer* following the determination that the 16S rRNA gene sequences of *E. adherens* and *Sinorhizobium* spp. placed the two genera within the same taxon (46). However, recent work indicates that sufficient genomic and phenotypic differences exist between symbiotic and non-symbiotic bacteria within *Ensifer* to warrant re-separation of the genus into *Sinorhizobium* (for the symbionts) and *Ensifer* (for the non-symbionts) (47–50). For these reasons, *Sinorhizobium* has been used in place of *Ensifer* throughout this paper.

### Core and symbiosis gene phylogenies and symbiosis island analysis

Sequences used for construction of the core genome phylogeny were a defined set of 120 protein coding genes previously shown to be present in >90% of bacterial genomes (51). This set had previously been used to identify proteins in representative members of the genus *Rhizobium* (21). The *Rhizobium* set was used to identify orthologous genes in representative strains from the genera *Mesorhizobium*, *Sinorhizobium*/*Ensifer*, and *Bradyrhizobium*. Sequences used for symbiosis gene phylogenies were identified by searching representative strains for genera-specific single-copy symbiosis genes, resulting in *Bradyrhizobium-*, *Mesorhizobium-*, *Sinorhizobium-*, and *Rhizobium*-specific symbiosis gene sets. Protein sequences from these sets were queried against each genome using TBLASTN (52) with a cutoff E-value of 1 × 10^−5^. Sequences were subsequently aligned with clustalo v 1.2.4 (53), concatenated, and a maximum likelihood phylogeny was constructed with RAxML v 8.2.1 (54). Trees were visualized using FigTree v 1.4.4 (https://github.com/rambaut/figtree/releases). To identify symbiosis islands, genomes were visually inspected for variations in GC content using the GC content toggle in the Graphs tab of Geneious Prime v 2024.0.7. Alignment of symbiosis islands was generated using DiGAlign v 2.0 (https://www.genome.jp/digalign/) (55).

## RESULTS AND DISCUSSION

### Updated taxonomy of Australian commercial inoculants

The genomes of 37 inoculant rhizobia strains were sequenced, and the sequences were submitted to the TYGS (https://tygs.dsmz.de/) for taxonomic analysis, along with the genomes of five previously sequenced strains: WSM1325 (24), CB782 (29), CC1192 (26), WSM1497 (27), and SU343 (28), which are the recommended inoculants for annual *Trifolium* spp., *Trifolium semipilosum*, *Cicer arietinum*, *Biserrula pelecinus*, and *Lotus corniculatus*, respectively. The 42 total sequences all matched known rhizobial genera, with 19 strains grouping within *Bradyrhizobium*, four within *Mesorhizobium*, one within *Methylobacterium*, 14 within *Rhizobium*, and four within *Sinorhizobium* (Table 2). Twenty-three of the submitted genomes met the species threshold with a known type strain (dDDH ≥70, ANI ≥96, AAI ≥96). For inoculant strains CB1717 and SU303, AAI values were greater than 96% for the closest TYGS match for *Bradyrhizobium huanghuaihaiense* CGMCC 1.10948^T^ and *Rhizobium laguerreae* FB206^T^, respectively, but dDDH and ANI values were both below the species thresholds for these type strains. Therefore, species names could not be definitively assigned for either strain, and instead, both strains were identified to the genus level as *Bradyrhizobium* sp. CB1717 and *Rhizobium* sp. SU303. Historical *Biserrula pelecinus* inoculant strain WSM1558 was not previously assigned to a species when its genome was reported in Colombi et al. (56). We found WSM1558 matched with *M. opportunistum* WSM2075^T^ slightly below the d_4_ dDDH threshold of 70, but both ANI and AAI values were above the species threshold for this type strain, suggesting that *M. opportunistum* was likely an appropriate species name for this strain. The remaining 19 inoculant strains did not match closely enough with a known type strain to be assigned a definitive species, and so were only classified to the genus level, with 10 *Bradyrhizobium*

**TABLE 2:** Summary of inoculant strain classification taxonomic updates to the commercial and historical rhizobial inoculants^*a*^

Species	Strain	Top TYGS subject	d_4_ dDDH [CI^*b*^]	ANI	AAI	Assembly
***B. arachidis***	**CB756**	***B. arachidis* LMG 26795^T^**	**87.7 [85.2–89.9]**	**98.4**	**98.7**	**ASM2105226v1**
***B. barranii***	**CC829**	***B. barranii* 144S4^T^**	**72.8 [69.8–75.6]**	**96.4**	**97.1**	**ASM2105236v1**
***B. barranii* subsp. *apii***	**CC1502**	***B. barranii* subsp. *apii* 38S5^T^**	**74.0 [70.9–76.8]**	**96.8**	**97.5**	**ASM2971422v1**
***B. brasilense***	**5G1B**	***B. brasilense* UFLA03-321^T^**	**70.2 [67.2–73.0]**	**96.3**	**97.2**	**ASM2971434v1**
***B. brasilense***	**CB627**	***B. brasilense* UFLA03-321^T^**	**93.4 [91.5–94.9]**	**98.9**	**99.0**	**ASM2971476v1**
***B. diazoefficiens***	**CB1809**	***B. diazoefficiens* USDA 110** ^**T**^	**89.4 [87.1–91.4]**	**98.7**	**99.0**	**ASM2105228v1**
***B. huanghuaihaiense***	**CB3035**	***B. huanghuaihaiense* CGMCC 1.10948^T^**	**77.7 [74.7–80.4]**	**97.4**	**98.0**	**ASM2520088v1**
***B. pachyrhizi***	**CB1923**	***B. pachyrhizi* PAC48^T^**	**84.4 [81.7–86.6]**	**98.0**	**98.1**	**ASM2971454v1**
***B. yuanmingense***	**CB1024**	***B. yuanmingense* CCBAU 10071^T^**	**81.9 [79.0–84.4]**	**97.9**	**98.3**	**ASM2520090v1**
*Bradyrhizobium* sp.	CB82	*B. centrolobii* BR 10245^T^	30.2 [27.8–32.7]	86.0	85.8	ASM2971440v1
*Bradyrhizobium* sp.	CB1015	*B. yuanmingense* CCBAU 10071^T^	52.4 [49.7–55.1]	93.6	94.6	ASM2520092v1
*Bradyrhizobium* sp.	CB1650	*B. neotropicale* BR 10247^T^	40.2 [37.7–42.7]	90.0	91.3	ASM2976191v1
*Bradyrhizobium* sp.	CB1717	*B. huanghuaihaiense* CGMCC 1.10948^T^	59.6 [56.8–62.4]	95.0	96.1	ASM2971432v1
*Bradyrhizobium* sp.	CB2312	*B. arachidis* LMG 26795^T^	53.8 [51.1–56.5]	93.9	95.3	ASM2971442v1
*Bradyrhizobium* sp.	CB3481	*B. hereditatis* WSM 1738^T^	33.4 [31.0–35.9]	87.6	90.3	ASM2971430v1
*Bradyrhizobium* sp.	CIAT3101	*B. rifense* CTAW71^T^	44.1 [41.5–46.6]	91.7	92.9	ASM2971494v1
*Bradyrhizobium* sp.	NC92	*B. glycinis* CNPSo 4016^T^	50.1 [47.5–52.7]	92.8	94.1	ASM2520086v1
*Bradyrhizobium* sp.	WSM471	*B. canariense* BTA-1^T^	57.7 [54.9–60.5]	94.4	95.6	ASM2105224v1
*Bradyrhizobium* sp.	WU425	*B. canariense* BTA-1^T^	57.3 [54.6–60.1]	94.4	95.9	ASM2105230v1
***M. ciceri***	**CC1192**	***M. ciceri* LMG 14989^T^**	**88.7 [86.2–90.7]**	**98.6**	**98.8**	**ASM161882v1**
***M. ciceri***	**WSM1497**	***M. ciceri* LMG 14989^T^**	**86.7 [84.1–88.9]**	**98.2**	**98.4**	**ASM167245v2**
***M. jarvisii***	**SU343**	***M. jarvisii* LMG 28313^T^**	**99.8 [99.6–99.9]**	**99.9**	**99.9**	**ASM1317086v1**
***M. opportunistum***	**WSM1558**	***M. opportunistum* WSM2075^T^**	**67.4 [64.5–70.3]**	**96.0**	**96.8**	**ASM2338000v1**
*Methylobacterium* sp.	CB376	*M. nodulans* ORS 2060^T^	28.8 [26.4–31.3]	85.6	83.2	ASM2971420v1
***R. hidalgonense***	**CB782**	***R. hidalgonense* FH14^T^**	**87.9 [85.4–90.0]**	**98.6**	**98.6**	**ASM52087v1**
***R. laguerreae***	**WSM1455**	***R. laguerreae* FB206^T^**	**85.0 [82.3–87.4]**	**98.4**	**98.7**	**ASM2105232v1**
***R. leguminosarum***	**CC283b**	***R. leguminosarum* USDA 2370** ^**T**^	**98.2 [97.3–98.7]**	**99.5**	**99.5**	**ASM2971424v1**
***R. ruizarguesonis***	**TA1**	***R. ruizarguesonis* UPM1133^T^**	**83.2 [80.4–85.7]**	**98.0**	**98.5**	**ASM2105242v1**
***R. sophoriradicis***	**CC511**	***R. sophoriradicis* CCBAU 03470^T^**	**84.6 [81.8–87.0]**	**98.3**	**98.6**	**ASM2520079v1**
***R. sullae***	**WSM1592**	***R. sullae* IS123^T^**	**89.0 [86.6–91.0]**	**98.4**	**98.6**	**ASM2520071v1**
*Rhizobium* sp.	CB3060	*R. leucaenae* USDA 9039^T^	38.7 [36.2–41.2]	89.6	92.6	ASM2520073v1
*Rhizobium* sp.	CB3090	*R. leucaenae* USDA 9039^T^	41.9 [39.4–44.4]	90.7	93.4	ASM2971428v1
*Rhizobium* sp.	CB3171	*R. leucaenae* USDA 9039^T^	38.5 [36.0–41.0]	89.5	92.6	ASM2971426v1
*Rhizobium* sp.	SRDI969	*R. ruizarguesonis* UPM1133^T^	55.1 [52.4–57.8]	94.0	95.6	ASM2515272v1
*Rhizobium* sp.	SU303	*R. laguerreae* FB206^T^	63.2 [60.3–66.0]	95.6	96.7	ASM2105234v1
*Rhizobium* sp.	WSM1274	*R. ruizarguesonis* UPM1133^T^	55.0 [52.2–57.7]	93.9	95.4	ASM2520077v1
*Rhizobium* sp.	WSM1325	*R. indicum* MCC 3961^T^	55.7 [53.0–58.4]	93.4	95.6	ASM2318v1
*Rhizobium* sp.	WSM4643	*R. ruizarguesonis* UPM1133^T^	54.7 [52.0–57.4]	94.0	95.4	ASM2515274v1
***S. medicae***	**SU277**	***S. medicae* USDA 1037^T^**	**98.3 [97.6–98.9]**	**99.7**	**99.7**	**ASM2520069v1**
***S. medicae***	**WSM1115**	***S. medicae* USDA 1037^T^**	**94.7 [93.0–95.9]**	**99.4**	**99.2**	**ASM2105256v1**
***S. meliloti***	**RRI128**	***S. meliloti* NBRC 14782^T^**	**90.1 [87.8–92.0]**	**98.5**	**98.7**	**ASM2105266v1**
***S. terangae***	**CB3126**	***S. terangae* USDA 4894^T^**	**91.6 [98.5–93.4]**	**98.7**	**98.6**	**ASM2971436v1**

^*a*^ Strains that match a TYGS subject are in bold; TYGS subjects reported as *Ensifer* have been changed to *Sinorhizobium* for clarity.

^*b*^ CI, confidence interval.

sp., one *Methylobacterium* sp., and eight *Rhizobium* sp. The high proportion (~45%) of inoculant strains that did not match a known type strain reflects the inherent diversity of these commercial strains.

### Replicon architecture and genomic location of symbiosis genes

Given the taxonomic diversity of inoculant rhizobia sequenced, we next wanted to understand the overall architecture of the inoculant genomes and compare them to the general replicon configuration across rhizobia genera. We also wanted to identify the genomic location of the symbiosis genes, with a particular focus on common nodulation (*nodABCIJ*) and nitrogen fixation (*nifHDKEN* and *fixABCX*) genes (Table S1), to better understand the potential of these genes to undergo HGT, generating novel rhizobia strains.

*Bradyrhizobium* spp. represent the largest group of commercial inoculants, with the 19 strains composed of three inoculants for temperate legumes (*B. barranii* CC829 for *Lotus peduculatus*, *Bradyrhizobium* sp. WU425 for *Lupinus* sp., and *Bradyrhizobium* sp. WSM471 for *Ornithopus* sp.) and 16 inoculants for tropical/subtropical legumes (including *B. diazoefficiens* CB1809 for *Glycine max*, *Bradyrhizobium* sp. NC92 for *Arachis hypogaea*, and *Bradyrhizobium* sp. CB1015 for *Vigna* sp.). Inoculant *Bradyrhizobium* strains sequenced here harbor a chromosome of ~7.8–9.8 Mb, similar to the well-studied *Glycine max* strain *B. diazoefficiens* USDA110^T^ (Table 3). *Bradyrhizobium* spp. are rarely reported to harbor plasmids (57, 58), and here, only three strains carry accessory plasmids (*B. barranii* CC829, *B. barranii* subsp. *apii* CC1502, and *Bradyrhizobium* sp. CB82). Strains of *B. barranii*, including the type strain *B. barranii* 144S4^T^, have between two and four plasmids, suggesting that multipartite genome architecture may be a characteristic of this species (59). Symbiosis genes in all inoculant *Bradyrhizobium* strains were also found chromosomally encoded within an SI, as is the case for well-studied model strain *B*.

**TABLE 3:** Genome structure of commercial and historical inoculant strains as well as select model organisms^*a*^

Strain	Genome size (Mbp)	Chromosome (Mbp)	Accessory replicons (Mbp)						
***Bradyrhizobium diazoefficiens* USDA 110^T^**	**9.10**	**9.10**							
*B. arachidis* CB756	9.82	9.82							
*B. barranii* CC829	10.5	9.63	pCC829_1 (0.43)	pCC829_2 (0.34)	pCC829_3 (0.11)				
*B. barranii* subsp. *apii* CC1502	10.2	9.76	pCC1502_1 (0.44)						
*B. brasilense* 5G1B	8.97	8.97							
*B. brasilense* CB627	9.15	9.15							
*B. diazoefficiens* CB1809	9.14	9.14							
*B. huanghuaihaiense* CB3035	9.61	9.61							
*B. pachyrhizi* CB1923	8.85	8.85							
*B. yuanmingense* CB1024	7.84	7.84							
*Bradyrhizobium* sp. CB82	10.1	9.24	pCB82_1 (0.90)						
*Bradyrhizobium* sp. CB1015	8.41	8.41							
*Bradyrhizobium* sp. CB1650	9.43	9.43							
*Bradyrhizobium* sp. CB1717	9.23	9.23							
*Bradyrhizobium* sp. CB2312	9.78	9.78							
*Bradyrhizobium* sp. CB3481	8.04	8.04							
*Bradyrhizobium* sp. CIAT3101	9.37	9.37							
*Bradyrhizobium* sp. NC92	8.36	8.36							
*Bradyrhizobium* sp. WSM471	7.79	7.79							
*Bradyrhizobium* sp. WU425	7.87	7.87							
***Mesorhizobium japonicum* R7A**	**6.53**	**6.53**							
*M. ciceri* CC1192	6.94	6.30	pMc1192 (0.65)						
*M. ciceri* WSM1497	7.20	6.66	pWSM1497 (0.53)						
*M. jarvisii* SU343	7.20	6.93	pMLSU343a (0.24)	pMLSU343b (0.02)					
*M. opportunistum* WSM1558	6.88	6.88							
***Methylobacterium nodulans* ORS 2060^T^**	**8.84**	**7.77**	**pMNOD01 (0.49**)	**pMNOD02 (0.46**)	**pMNOD03 (0.04**)	**pMNOD04 (0.04**)	**pMNOD05 (0.02**)	**pMNOD06 (0.01**)	**pMNOD07 (0.01**)
*Methylobacterium* sp. CB376	7.69	7.64	pCB376_1 (0.06)						
***Rhizobium johnstonii* 3841^T^**	**7.75**	**5.06**	**pRL12 (0.87**)	**pRL11 (0.68**)	**pRL10 (0.49**)	**pRL9 (0.35**)	**pRL8 (0.15**)	**pRL7 (0.15**)	
*R. hidalgonense* CB782	6.70	4.38	pCB782_1 (1.56)	pCB782_2 (0.51)	pCB782_3 (0.25)				
*R. laguerreae* WSM1455	6.97	4.82	pWSM1455_1 (1.06)	pWSM1455_2 (0.52)	pWSM1455_3 (0.29)	pWSM1455_4 (0.28)			
*R. leguminosarum* CC283b	8.06	5.07	pCC283b_1 (1.16)	pCC283b_2 (0.60)	pCC283b_3 (0.56)	pCC283b_4 (0.55)	pCC283b_5 (0.13)		
*R. ruizarguesonis* TA1	7.62	5.04	pTA1_1 (0.81)	pTA1_2 (0.66)	pTA1_3 (0.61)	pTA1_4 (0.50)			
*R. sophoriradicis* CC511	6.97	4.50	pCC511_1 (0.76)	pCC511_2 (0.65)	pCC511_3 (0.47)	pCC511_4 (0.40)	pCC511_5 (0.19)		
*R. sullae* WSM1592	7.60	4.14	pWSM1592_1 (2.76)	pWSM1592_2 (0.47)	pWSM1592_3 (0.23)				
*Rhizobium* sp. CB3060	7.28	4.07	pCB3060_1 (2.26)	pCB3060_2 (0.54)	pCB3060_3 (0.28)	pCB3060_4 (0.14)			
*Rhizobium* sp. CB3090	6.49	3.91	pCB3090_1 (1.77)	pCB3090_2 (0.56)	pCB3090_3 (0.24)				
*Rhizobium* sp. CB3171	7.88	3.97	pCB3171_1 (2.83)	pCB3171_2 (0.54)	pCB3171_3 (0.21)	pCB3171_4 (0.19)	pCB3171_5 (0.10)	pCB3171_6 (0.035)	
*Rhizobium* sp. SRDI969	6.84	4.90	pSRDI969_1 (0.64)	pSRDI969_2 (0.55)	pSRDI969_3 (0.43)	pSRDI969_4 (0.33)			
*Rhizobium* sp. SU303	7.07	5.01	pSU303_1 (0.94)	pSU303_2 (0.53)	pSU303_3 (0.33)	pSU303_4 (0.26)			
*Rhizobium* sp. WSM1274	7.08	4.91	pWSM1274_1 (1.18)	pWSM1274_2 (0.42)	pWSM1274_3 (0.31)	pWSM1274_4 (0.25)			
*Rhizobium* sp. WSM1325	7.42	4.77	pR132501 (0.83)	pR132502 (0.66)	pR132503 (0.52)	pR132504 (0.35)	pR132505 (0.29)		
*Rhizobium* sp. WSM4643	6.44	4.86	pWSM4643_1 (0.63)	pWSM4643_2 (0.55)	pWSM4643_3 (0.41)				
***Sinorhizobium meliloti* 1021**	**6.69**	**3.65**	**pSymB (1.68**)	**pSymA (1.35**)					
*S. medicae* SU277	6.76	3.75	pSU277_1 (1.53)	pSU277_2 (1.02)	pSU277_3 (0.37)	pSU277_4 (0.07)			
*S. medicae* WSM1115	7.06	4.10	pWSM1115_1 (1.55)	pWSM1115_2 (1.13)	pWSM1115_4 (0.28)				
*S. meliloti* RRI128	7.27	3.73	pRRI128_1 (1.61)	pRRI128_2 (1.29)	pRRI128_3 (0.31)	pRRI128_4 (0.18)	pRRI128_5 (0.15)		
*S. terangae* CB3126	6.63	3.97	pCB3126_1 (2.09)	pCB3126_2 (0.57)					

^*a*^ Well-studied model organisms are in bold; replicons with gray shading contain major symbiosis genes.

*diazoefficiens* USDA110^T^ (60). SIs are commonly found in *Bradyrhizobium* genomes and have a range of characteristics suggesting they may have been acquired by HGT, including a lower GC content and a different codon usage compared to non-SI genes (61). SIs were identified in every inoculant *Bradyrhizobium* genome sequence and are highly variable in size, ranging from 0.49 to 1.2 Mbp with an average size of 0.79 Mbp (Table 4). In most cases, the SIs were flanked by a tRNA gene at one end, and a recombinase family protein encoding gene at the other. Four strains had atypical SI borders, with *B. brasilense* 5G1B and *B. huanghuaihaiense* CB3035 containing a helicase-related protein, and *Bradyrhizobium* sp. CB1650 containing a DDE-type integrase/transposase/recombinase instead of the characteristic recombinase family protein, while *Bradyrhizobium* sp. CB3481 was missing both typical markers; instead, the SI was bordered by a transposase and a tyrosine-type recombinase/integrase (Table 4). *Bradyrhizobium* SIs have been previously reported to be divided across three loci, labeled as regions A, B, and C (58, 62). While regions corresponding to SI A were readily identifiable (Table 4), loci corresponding to regions B and C could not be conclusively identified in many of the *Bradyrhizobium* inoculant genomes (Table S2), indicating that a variation in SI number and configuration exists across this genus.

**TABLE 4:** Size, GC content, and flanking loci of SIs within the chromosome of the *Bradyrhizobium* sp. inoculant genomes^*a*^

Strain	Genome size (bp)	Chromosome GC content (%)	SI size (bp)	SI GC content (%)	Left flank (locus tag)	Right flank (locus tag)
***B. diazoefficiens* USDA110^T^**	**9,105,828**	**64.1**	**682,397**	**59.4**	**Recombinase (BJA_RS07875**)	**tRNA-Val (BJA_RS10650**)
*B. barranii* CC829	10,502,301	63.6	485,737	59.9	Recombinase (BjapCC829_RS06045)	tRNA-Val (BjapCC829_RS08225)
*B. diazoefficiens* CB1809	9,136,715	64.0	672,335	59.4	tRNA-Val (BdzoCB1809_RS36155)	Recombinase (BdzoCB1809_RS39005)
*Bradyrhizobium* sp. WU425	7,868,972	63.3	569,581	59.5	tRNA-Val (BcanWU425_RS31625)	Recombinase (BcanWU425_RS34010)
*Bradyrhizobium* sp. CB1015	8,412,734	63.8	577,690	58.9	tRNA-Val (N2604_RS05620)	Recombinase (N2604_RS08035)
*B. yuanmingense* CB1024	7,840,305	63.9	845,148	58.6	tRNA-Ile (N2605_RS25055)	Recombinase (N2605_RS28330)
*B. arachidis* CB756	9,825,352	63.6	881,791	58.1	Recombinase (BaraCB756_RS07940)	tRNA-Ile (BaraCB756_RS11540)
*Bradyrhizobium* sp. NC92	8,355,732	63.9	844,827	58.4	tRNA-Ile (N2602_RS31355)	Recombinase (N2602_RS34865)
*Bradyrhizobium* sp. WSM471	7,785,529	63.4	569,250	59.1	Recombinase (BcanWSM471_RS06555)	tRNA-Val (BcanWSM471_RS09065)
*B. brasilense* 5G1B	8,976,199	63.8	1,186,148	60.3	Helicase-related protein (QA635_RS30235)	tRNA-Glu (QA635_RS35330)
*Bradyrhizobium* sp. CB82	10,142,525	62.5	1,005,155	58.6	tRNA-Val (QA640_RS35850)	Recombinase (QA640_RS40010)
*B. brasilense* CB627	9,148,457	63.7	823,972	58.6	Recombinase (QA636_RS33020)	tRNA-Glu (QA636_RS36455)
*Bradyrhizobium* sp. CB1650	9,430,299	63.1	1,098,987	59.1	tRNA-Val (QA641_RS35410)	DDE-type integrase/transposase/recombinase (QA641_RS40185)
*Bradyrhizobium* sp. CB1717	9,232,581	63.8	647,706	58.6	tRNA-Ile (QA649_RS34405)	Recombinase (QA649_RS37030)
*B. pachyrhizi* CB1923	8,851,675	63.8	636,927	58.9	tRNA-Val (QA639_RS33045)	Recombinase (QA639_RS35855)
*Bradyrhizobium* sp. CB2312	9,780,375	63.4	899,892	58.6	tRNA-Ile (QA642_RS38195)	Recombinase (QA642_RS41855)
*B. huanghuaihaiense* CB3035	9,614,050	63.8	1,208,964	59.1	tRNA-Val (N2603_RS35765)	Helicase-related protein (N2603_RS41080)
*Bradyrhizobium* sp. CB3481	8,035,627	62.6	772,783	58.7	Transposase (QA643_RS25030)	Tyrosine-type recombinase/integrase (QA643_RS28720)
*B. barranii* subsp. *apii* CC1502	10,198,616	63.5	761,276	59.6	Recombinase (QA633_RS06225)	tRNA-Val (QA633_RS09570)
*Bradyrhizobium* sp. CIAT3101	9,368,452	63.4	605,622	57.8	Hypothetical protein (QA645_RS38425)	tRNA-Ile (QA645_RS40690)
**Inoculant average**	9,079,289.26	63.52	794,410.05	58.94		

^*a*^ Model strain *B. diazoefficiens* USDA110^T^ is included for reference in bold.

The *Mesorhizobium* spp. inoculants for *Cicer arietinum* (*M. ciceri* CC1192), *Biserrula pelecinus* (*M. ciceri* WSM1497 and *M. opportunistum* WSM1558), and *Lotus corniculatus* (*M. jarvisii* SU343) all contain chromosomes of similar size (6.29–6.95 Mbp), with symbiosis genes inside ICEs on the chromosomes (Table 2). These elements are either monopartite (CC1192) or tripartite (WSM1497 and SU343) in structure, as has been previously reported (26–28). WSM1558 also contains a tripartite ICE with a total size of 619.9 kb comprising α (6,169,134 to 6,756,269 bp), β (2,982,080 to 2,997,617 bp), and γ (2,736,515 to 2,758,719 bp) regions. CC1192, WSM1497, and SU343 also possess RepABC-type accessory plasmids, which are absent in WSM1558 and model strain *M. japonicum* R7A (Table 3). The CC1192 plasmid does encode some recognized symbiosis genes (*fixNOQP* and *fixGHI*), but these are not essential to symbiosis (9), likely due to additional copies of these genes encoded on the CC1192 ICE. No plasmid-encoded symbiosis genes were identified on the plasmids of either WSM1497 or SU343.

The genome of *Methylobacterium* sp. CB376 consists of a chromosome and a plasmid, with the symbiosis genes encoded from the chromosome. While the symbiosis genes are in relatively close proximity, the overall region of ~330 kb (% GC 72.5) does not exhibit the consistent low GC content characteristic of an SI. Closer inspection of the ~6.5 kb region containing nodulation genes *nodD* and *nodABCIJH* showed it had a GC content of 52.5% compared to a chromosomal GC content of 71.6%. Model strain *M. nodulans* ORS 2060^T^ also encodes its symbiosis genes on the chromosome but carries seven additional plasmids (Table 3), which amount to an additional 1.14 Mbp size difference between the two *Methylobacterium* spp.

**Fig 1:**
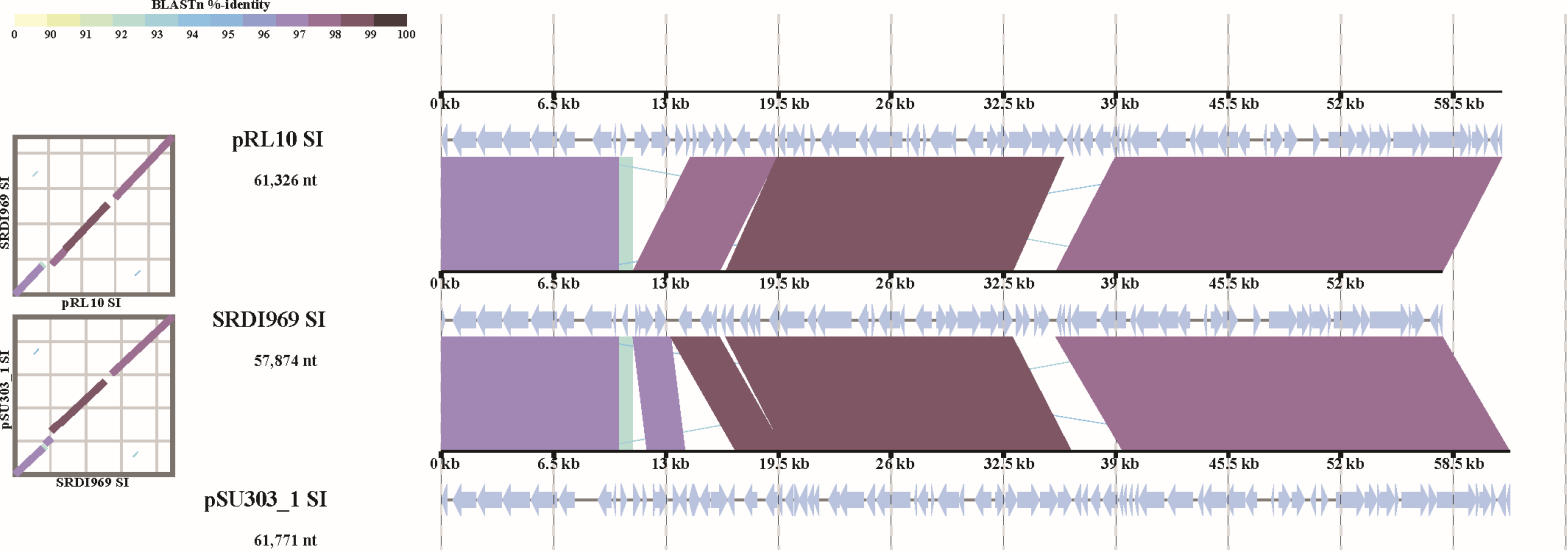
Comparison of SIs from *Rhizobium* sp. SRDI969, *Rhizobium* sp. SU303, and *R*. *johnstonii* 3841^T^. Dot plots are shown on the left, and alignments are shown on the right.

Genomes of members of *Rhizobium* are generally composed of a chromosome and several plasmids of varying sizes (1), such as the well-studied strain *R. johnstonii* 3841^T^, which contains a 5.06 Mbp chromosome and six plasmids between 0.15 and 0.87 Mbp. Symbiosis genes in *Rhizobium* spp. are usually encoded on one of these plasmids (designated pSym), which for 3841 is pRL10 (63). Genomes of the *Rhizobium* inoculants sequenced in this study similarly contained between two and six plasmids ranging in size from 0.035 to 2.83 Mbp, with an average size of 0.66 Mbp. Symbiosis genes are encoded on a single plasmid (pSym) for 13 of the 14 *Rhizobium* inoculant strains. The exception was for the *Vicia faba* inoculant *Rhizobium* sp. SRDI969, where the symbiosis genes are unusually encoded on the chromosome in a ~ 75 kb region, as recently reported (64) (Table 3). Comparison of the symbiosis region of *Rhizobium* sp. SRDI969 to plasmid-borne symbiosis gene regions from other *Vicia*/*Pisum*/*Lens*-nodulating strains, *Rhizobium* sp. SU303 (on pSU303_1) and *R. johnstonii* 3841^T^ (pRL10), showed all three regions shared a highly similar overall gene arrangement over ~57 kb and >97% nucleotide identity between coding sequences (Fig. 1). All three regions exhibited a GC content of between 56.2% and 56.3%; the plasmid-borne regions are flanked by transposase encoding genes while the chromosomal region has a transposase on one side and *rpoN* encoded on the other. This suggests that the chromosomal SRDI969 sym genes may have resulted from a transfer event from a pSU303_1 or pRL10-like plasmid to the SRDI969 chromosome. Inter-replicon symbiosis gene transfer has been documented (65), and Mazurier and Laguerre reported *nod* and *nif* genes in lentil-nodulating *R. leguminosarum* strains were localized on either the chromosome or a large extrachromosomal replicon (66). It is therefore possible that other *Rhizobium* strains may encode their symbiosis genes chromosomally, like SRDI969.

Genomes of *Sinorhizobium* inoculants for *Medicago* spp. (*S. meliloti* RRI128 and *S. medicae* WSM1115), *Trigonella foenum-graecum* (*S. medicae* SU277), and *Desmanthus* (*S. terangae* CB3126) all have two large megaplasmid replicons, with symbiosis genes present on the smaller of the two (Table 3), as is the case with model strain *S. meliloti* 1021 (also referred to as Sm1021) (67). In Sm1021, the largest megaplasmid (pSymB) is considered a hybrid replicon with features of both chromosomes and plasmids, called a chromid (68). This classification is, in large part, due to essential protein synthesis genes *engA* and tRNA-Arg, which are located on pSymB in Sm1021 (69) but typically found on chromosomes in other bacteria (51). Consistent with Sm1021, replicons pRRI128_1 (*S. meliloti* RRI128), pWSM1115_1 (*S. medicae* WSM1115), and pSU277_1 (*S. medicae* SU277) each encode *engA* and tRNA-Arg. These replicons also have a GC content more similar to that of their corresponding chromosomes and encode plasmid-like RepABC-type replication systems, suggesting that they, like pSymB, may be chromids. Interestingly, neither *engA*, tRNA-Arg, nor any other essential genes described by Parks et al. (51) were found on plasmids pCB3126_1 or pCB3126_2 of *S. terangae* CB3126; they are instead encoded from the chromosome, suggesting chromids may not be a conserved feature of this species’ genome. *S. meliloti* RRI128, *S. medicae* WSM1115, and *S. medicae* SU277 also carry one to three additional plasmids, which are absent in Sm1021 and CB3126 (Table 3). These accessory or “cryptic” plasmids have been previously observed in other *Sinorhizobium* spp. (70–72), but their specific roles remain unknown (19).

### Inoculant core and symbiosis gene phylogenies and origin of strains isolated from Australia

With an understanding of the identity and genomics of the Australian commercial inoculants, we next phylogenetically compared strains from the four significant inoculant genera to other well-studied rhizobia for which complete genome sequences were available. This comparison was performed on core and symbiosis gene sets, to explore the relationship between inoculant strains and other rhizobia and detect potential instances of symbiosis gene transfer.

#### Mesorhizobium

**Fig 2:**
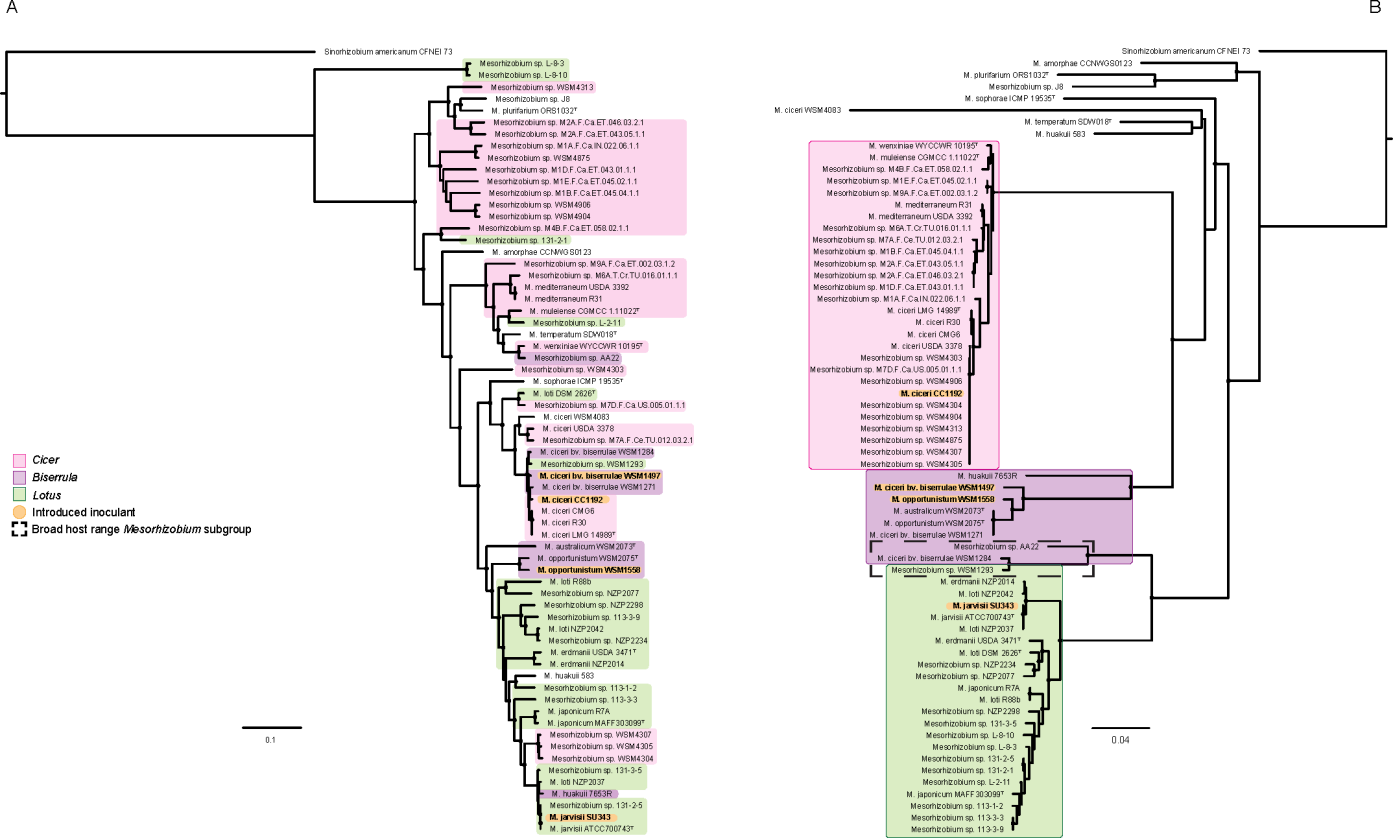
Core (**A**) and symbiosis (**B**) gene phylogenies of the genus *Mesorhizobium*. Strains are overlayed with their host legume of isolation using the colors pink, purple, and green to represent *Cicer*, *Biserrula*, and *Lotus*, respectively. Trees were constructed using RAxML with *Sinorhizobium americanum* CFNEI 73 set as the outgroup. Nodes with 100% bootstrap support are marked with a black circle.

The four sequenced *Mesorhizobium* inoculant strains were phylogenetically compared to 54 strains of *Mesorhizobium* at both the core and the symbiosis gene levels (Fig. 2). Strains isolated from *Biserrula*, *Cicer*, and *Lotus* were interspersed in the core genome tree, in contrast to the symbiosis tree where they clustered into three host-based groups (Fig. 2). The high level of discordance between these two phylogenies is characteristic of the frequently observed horizontal transfer of symbiosis genes encoded on *Mesorhizobium* symbiosis ICEs (73). All four *Mesorhizobium* inoculant strains are known to have been introduced into Australia, and the core genome phylogeny of *M. ciceri* CC1192 (originating from Israel [74]) and *M. ciceri* WSM1497 (Greece [75]) largely reflects this, with both grouping with other strains from the Mediterranean. *M. jarvisii* SU343 from the USA (76) clustered most closely with *Lotus*-nodulating strains isolated from Japan and New Zealand. *Lotus* spp. are native to Japan and the USA, but not to New Zealand, where *Lotus* spp. are introduced forage legumes (77). *Biserrula* inoculant strain *M. opportunistum* WSM1558 isolated from Italy (78) grouped most closely with *M. opportunistum* WSM2075^T^ isolated from Australia. WSM2075, along with *M. australicum* WSM2073^T^, has previously been shown to have acquired the symbiosis ICE from *M. ciceri* WSM1271, a *Biserrula* strain introduced from Italy to Australia in 1995 (79–81). Both *M. australicum* and *M. opportunistum* are present in Australian soils, as non-symbiotic strains (i.e., those lacking any discernible symbiosis genes) were recently isolated from cultivated and uncultivated Western Australian soils (56). In addition to Italy and Australia, strains of *M. opportunistum* have been isolated from the nodules of *C. arietinum* from Portugal (82) and *C. canariense* from the Canary Islands (83), suggesting that *M. opportunistum* is a widely distributed species capable of accepting symbiosis ICEs.

Ten strains appear to contain near-identical symbiosis genes to *M. ciceri* CC1192 despite different genetic backgrounds (Fig. 2). Eight of these strains (WSM4303 through WSM4906) are known recipients of ICE*Mc*Sym^1192^ that were recently isolated from Australian soils (9, 10). Additionally, genomic analysis of *Mesorhizobium* sp. M7D.F.Ca.US.005.01.1.1 and *M. ciceri* USDA 3378 shows that symbiosis ICE from CC1192 aligns to M7D.F.Ca.US.005.01.1.1 with 99.997% pairwise identity across a single region, and to USDA 3378 with 89.1% pairwise identity across 17 contigs, suggesting that they too share a ICE*Mc*Sym^1192^ with *M. ciceri* CC1192. *Mesorhizobium* sp. M7D.F.Ca.US.005.01.1.1 was isolated from Washington, USA, in 2013 (84) and has a core genome more similar to *M. loti* DSM 2626^T^, while USDA 3378 was also isolated from the USA, but in 1940 (Rhizobium Dataset, USDA-ARS GRIN, https://www.ars-grin.gov/Rhizobium).

#### Sinorhizobium

**Fig 3:**
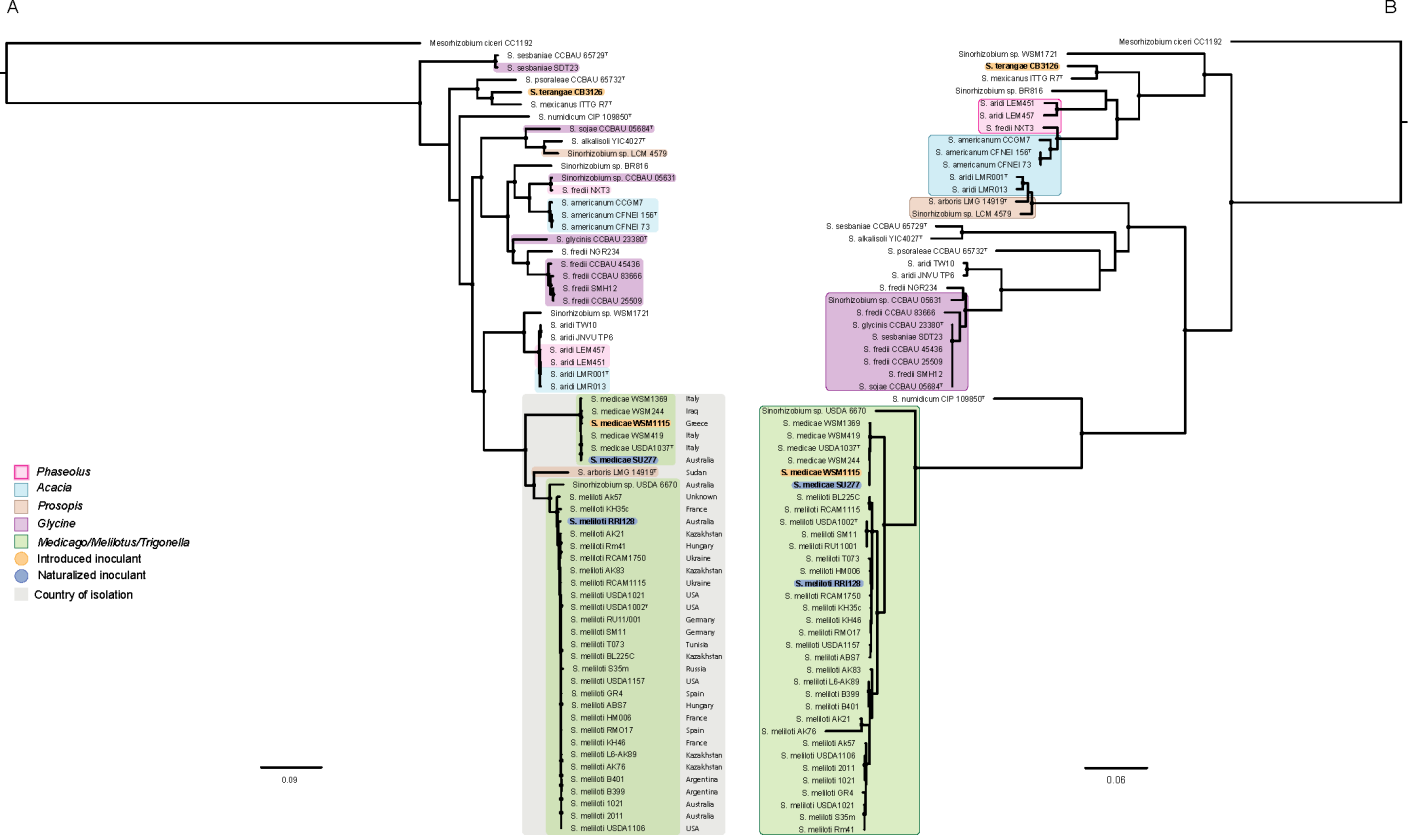
Core (**A**) and symbiosis (**B**) gene phylogenies of the genus *Sinorhizobium*. Strains are overlayed with their host legume of isolation using the colors pink, blue, brown, purple, and green to represent *Phaseolus*, *Acacia*, *Prosopis*, *Glycine*, and *Medicago*, *Melilotus*, and *Trigonella*, respectively. Select strains within the core genome phylogeny are overlayed with a gray box in which the country of isolation is indicated. Trees were constructed using RAxML with *Mesorhizobium ciceri* CC1192 set as the outgroup. Nodes with 100% bootstrap support are marked with a black circle.

In contrast to the high discordance of the *Mesorhizobium* strains, core and symbiosis gene phylogenies of inoculant *Sinorhizobium* and the 62 selected genomes showed a relatively low level of discordance (Fig. 3). Two of the four inoculant strains are exotic (*S. medicae* WSM1115 from Greece [3] and *S. terangae* CB3126 from Mexico [85]), and both cluster closely to other strains from these areas on core and symbiosis gene trees. The remaining strains, *S. medicae* SU277 for *Trigonella foenum-graecum* and *S. meliloti* RRI128 for *Medicago* spp., were both isolated from Australia (86, 87). The *S. medicae* and *S. meliloti* clades on the core gene tree are in proximity (Fig. 3), which is consistent with previous reports describing the geographic origins as well as the close phylogenetic and symbiotic relationship between these species (19, 88–91). *S. medicae* SU277 is similar to strains isolated from Greece, Italy, and Iraq, suggesting that it may have originated from the Mediterranean Basin and/or the Fertile Crescent region. Additionally, *S. meliloti* RRI128 is similar to many strains from Europe and the Americas but appears most similar to strains isolated in Kazakhstan, Ukraine, and Hungary, suggesting it may have originated from eastern Europe (Fig. 3). This is consistent with the proposed center of *Medicago* diversity in the Caucasus region (89). Importantly, the only available genome sequence of a *Sinorhizobium* strain isolated from an Australian native legume (*Indigofera* sp.)*—Sinorhizobium* sp. WSM1721 (92)—does not cluster with the introduced inoculant strains, further supporting the likely exotic origin of the inoculant strains isolated from Australia.

#### Rhizobium

**Fig 4:**
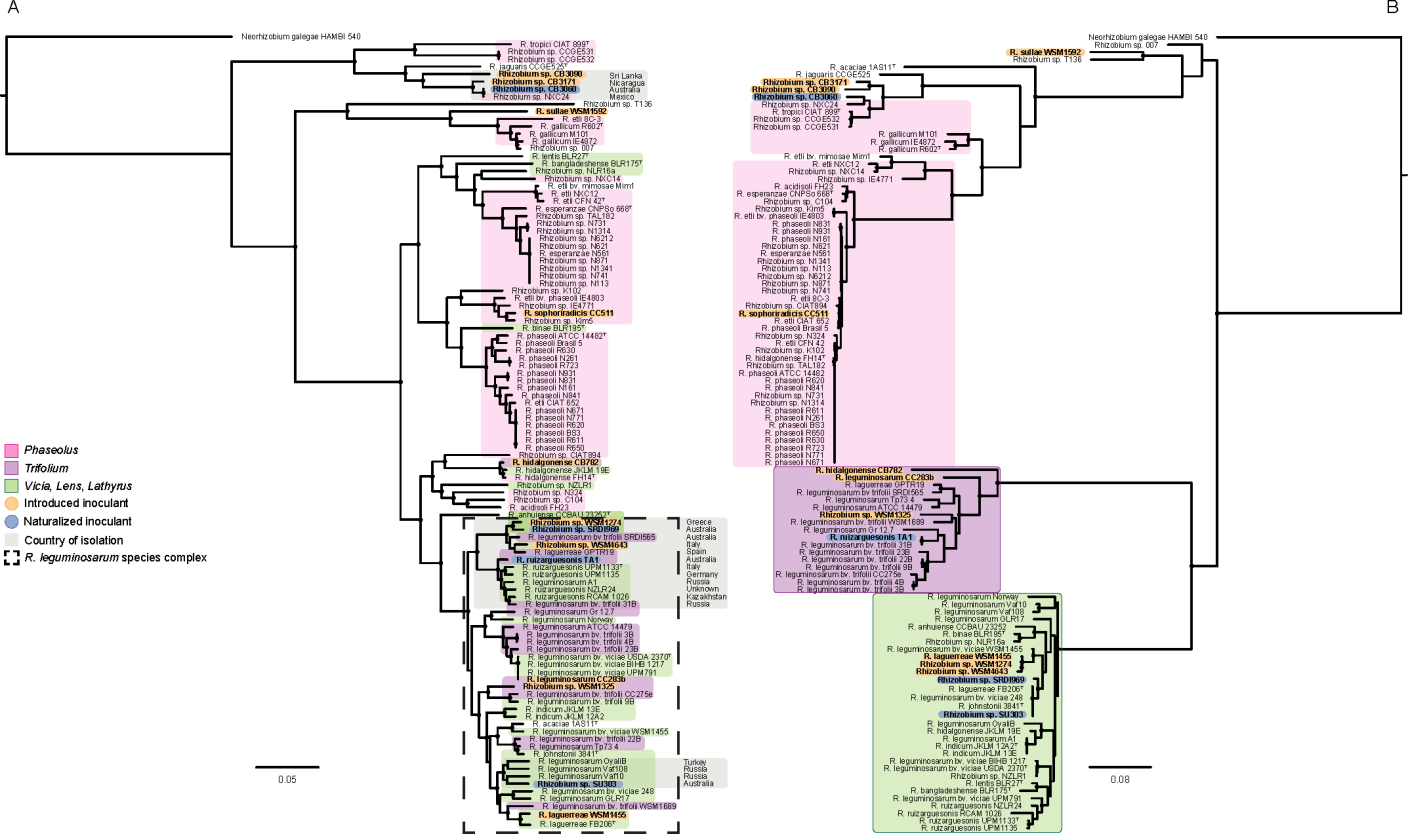
Core (**A**) and symbiosis (**B**) gene phylogenies of the genus *Rhizobium*. Strains are overlayed with their host legume of isolation using the colors pink, purple, and green to represent *Phaseolus*, *Trifolium*, and *Vicia*, *Lens*, and *Lathyrus*, respectively. Select strains within the core genome phylogeny are overlayed with a gray box in which the country of isolation is indicated. Trees were constructed using RAxML with *Neorhizobium galegae* HAMBI 540 set as the outgroup. Nodes with 100% bootstrap support are marked with a black circle.

The 14 *Rhizobium* inoculants are widely dispersed throughout the core genome phylogeny (Fig. 4), which is consistent with their taxonomic assignments (Table 2). Eight of these strains that nodulate either *Trifolium* or *Vicia*, *Lens*, and *Lathyrus* fall within the *Rhizobium leguminosarum* species complex (Rlc), which is a cluster of related strains representing at least 18 clades, each of which could be considered a distinct species (21, 93). Five of these strains were introduced as inoculants: *Rhizobium* sp. WSM1274 and *R. laguerreae* WSM1455 for *Vicia faba* from Greece, *Rhizobium* sp. WSM4643 for *Pisum* and *Vicia* from Italy, *R. leguminosarum* CC283b for *Trifolium ambiguum* from Russia, and *Rhizobium* sp. WSM1325 for a range of annual *Trifolium* spp. from Greece (5, 94–96). The core genomes of the Australian isolated strains *Rhizobium* sp. SRDI969 for *Vicia faba*, *R. ruizarguesonis* TA1 for *Trifolium*, and *Rhizobium* sp. SU303 for *Lathyrus* clustered with other strains from across Europe (Fig. 4), suggesting that these strains may have originated from Southern or Eastern Europe.

On the symbiosis gene tree, the inoculants and other strains within the Rlc cluster into two clear clades based on their host of isolation (Fig. 4). This separation is consistent with known host-strain specificities for these strains, as *Rhizobium* spp. that nodulate *Trifolium* or *Vicia*, *Lens*, and *Lathyrus* are known to be specific to those hosts (97). Strain TA1, originally isolated from Tasmania, is symbiotically nearly identical to *R. leguminosarum* 31B from Russia. Inoculant strains *Rhizobium* sp. SRDI969 and *Rhizobium* sp. SU303 both grouped with strains from the UK (248 and 3841) (98) and Tunisia (FB206) (99). Given that both the core and the symbiosis genes match closely with strains isolated overseas, as well as the geographical isolation of Tasmania and the lack of native *Trifolium* spp. prior to European colonization, it is highly likely that SRDI969, TA1, and SU303 are exotic strains that were introduced into Australia post-European colonization.

Six inoculant strains are located outside the Rlc group on the core gene phylogeny. *R. sullae* WSM1592, the inoculant for the temperate legume *Hedysarum coronarium*, was isolated from Italy (25) and grouped separately on both the core and the symbiosis gene trees from other characterized strains, being most closely related to strains of *R. gallium* isolated from France, Spain, Mexico, and Canada (100, 101). *R. sophoriradicis* CC511, the inoculant for *Phaseolus* spp. isolated from the USA (102), groups with other *Phaseolus*-nodulating *Rhizobium* on both the core and the symbiosis gene trees. Although *R. hidalgonense* CB782, the inoculant strain from Kenya for sub-tropical *Trifolium semipilosum*, did not cluster with other temperate *Trifolium*-nodulating strains within the Rlc on the core genome tree, it did group with these strains on the symbiosis tree. For *Rhizobium* sp. CB3060, the inoculant strain for the tropical legume *Leucaena leucocephala* isolated from north-eastern Australia, both the core and the symbiosis genes align closely with inoculant strain *Rhizobium* sp. CB3090 for *Gliricidia* spp. from Sri Lanka and *Calliandra* spp. inoculant *Rhizobium* sp. CB3171 from Nicaragua, as well as the *Phaseolus*-nodulating strain *Rhizobium* sp. NXC24 from Mexico. This suggests *Rhizobium* sp. CB3060 may have originated from Central America, which is the native origin of *L. leuococephala*, and have been introduced when the legume was brought to northern Australia in the 1890s (103).

Strain *R. laguerreae* WSM1455 was a previously long-standing commercial inoculant strain for *Vicia faba* between 2002 and 2022, and was originally isolated from *V. faba* in Greece (3, 5). The core genome of this strain groups with *R. laguerreae* FB206^T^ from Tunisia, while its symbiosis genes group with the historical inoculant for *V. faba*, WSM1274 from Greece and the current *Pisum*, *Lens*, and *Lathyrus* inoculant strain *Rhizobium* sp. WSM4643 from Italy (Fig. 4). An incomplete genome sequence was submitted to NCBI in June of 2012, under the name *R. leguminosarum* bv. *viciae* WSM1455 (GCF_000271805.1). However, the chromosome sequence of GCF_000271805.1 clusters with *R. acaciae*, while the symbiosis genes cluster with *R. laguerreae* WSM1455, *Rhizobium* sp. WSM1274, and *Rhizobium* sp. WSM4643. The *R. laguerreae* WSM1455 and *R. acaciae* GCF_000271805.1 sequences share an ANI value of only 94.3%. The original source material for *R. acaciae* GCF_000271805.1 is no longer available, therefore it was not possible to verify why this sequence is so different from the *R. laguerreae* WSM1455 reported here. Nevertheless, the *R. laguerreae* WSM1455 strain sequenced in this work was sourced directly from AIRG, which houses the Australian mother culture collection that is supplied to inoculant manufacturers, and therefore represents the genome of this commercial inoculant strain.

Discordance between core and symbiosis gene phylogenies has been noted previously (104) and is likely strongly influenced by conjugative transfer of symbiosis plasmids between *Rhizobium* strains (105, 106). There is discordance between the core and the symbiosis gene trees. However, the strains that nodulate *Phaseolus* are not intermingled with the *Trifolium* and the *Vicia*, *Lens*, and *Lathyrus* rhizobia of the Rlc on the core gene tree (Fig. 4). This is probably due to *Pisum*, *Lens*, *Vicia* spp. and many *Trifolium* spp. having centers of origin in Europe or western Asia (107–111), in contrast to *Phaseolus* bean, which was introduced more recently to this region from the Americas (112). This introduction likely brought *Phaseolus* bean-nodulating rhizobia in plant matter, seed, or soil, as has been suggested by García-Fraile et al. (113). That *Phaseolus* bean-nodulating rhizobia were geographically separated from other *Rhizobium* spp. for much of their evolutionary histories may contribute to the apparent separation of *Phaseolus*-nodulating rhizobia from the *Rhizobium* within the Rlc.

#### Bradyrhizobium

**Fig 5:**
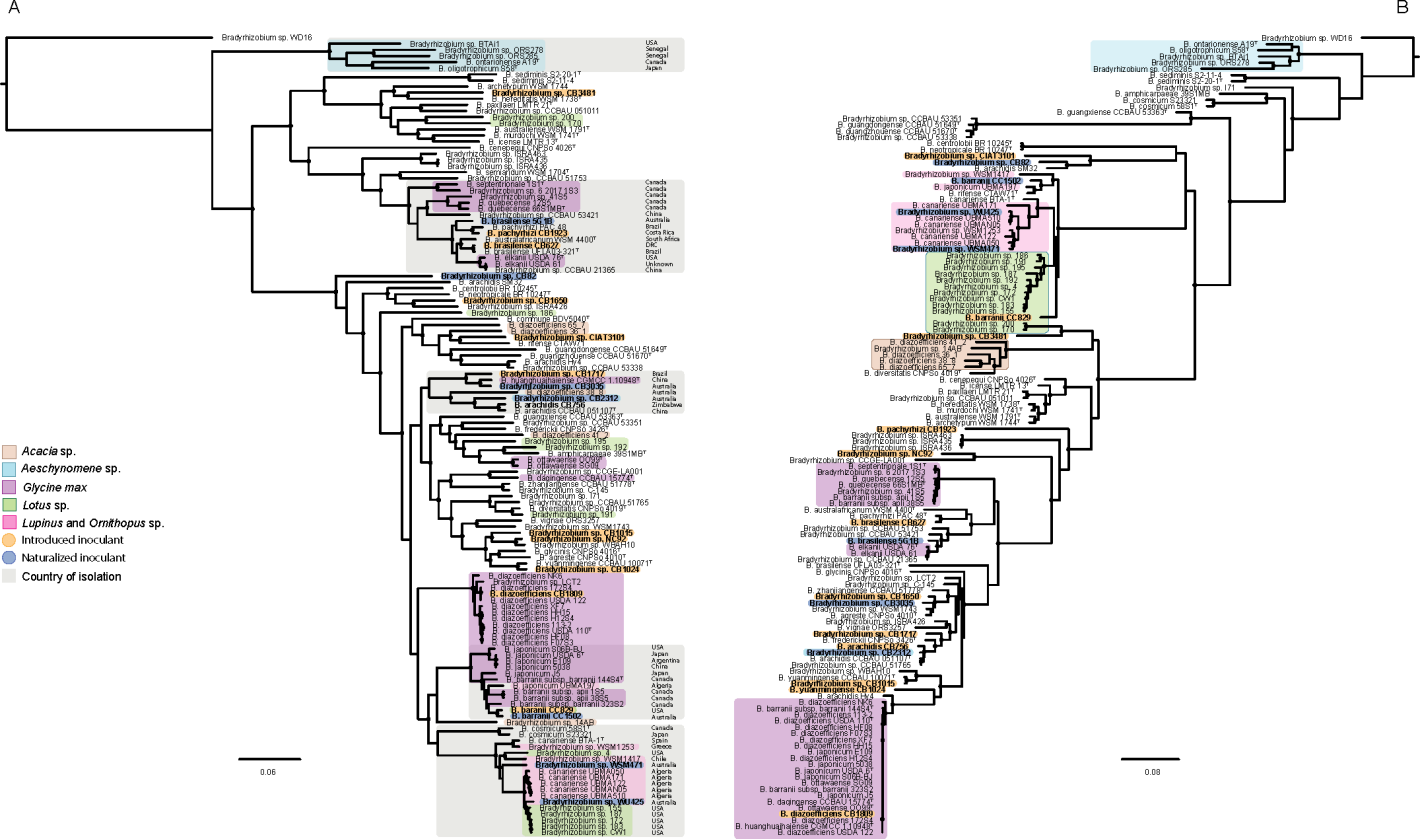
Core (**A**) and symbiosis (**B**) gene phylogenies of the genus *Bradyrhizobium*. Strains are overlayed with their host legume of isolation using the colors brown, blue, purple, green, and pink to represent *Acacia*, *Aeschynomene*, *Glycine max*, *Lotus*, and *Lupinus* and *Ornithopus*, respectively. Select strains within the core genome phylogeny are overlayed with a gray box in which the country of isolation is indicated. Trees were constructed using RAxML with *Bradyrhizobium* sp. WD16 set as the outgroup. Nodes with 100% bootstrap support are marked with a black circle. Strains *B. commune* BDV5040^T^ and *B. semiaridum* WSM 1704^T^ are missing several symbiosis genes used for alignment construction and were therefore left off the symbiosis gene tree.

Comparison of the 19 *Bradyrhizobium* inoculant strains to 127 sequenced *Bradyrhizobium* strains showed they were widely dispersed throughout the core gene tree (Fig. 5), indicating a high level of diversity among the commercial *Bradyrhizobium* inoculants. Even among seven *Bradyrhizobium* inoculant strains isolated from Australia, there was a significant diversity, with these strains belonging to six different species. Three strains are inoculants for temperate legumes, and the remaining four nodulate tropical legumes. Both *Bradyrhizobium* sp. WSM471 and WU425 are inoculant strains for *Ornithopus* and *Lupinus*. These strains are closely related to *B. canariense* BTA-1^T^ (Spain) and *Bradyrhizobium* sp. WSM1253 (Greece) on the core genome tree, and they cluster in a closely related clade on the symbiosis tree (Fig. 5). Both *Ornithopus* and *Lupinus* spp. are introduced legume genera that have been grown in Australia for over a century. Initially, these legumes required inoculation with compatible rhizobia; however, over time, compatible strains have become established in Australian soils (114). Both WSM471 and WU425 are therefore exotic strains of European origin, which is consistent with a previous report based on multilocus sequence typing (MLST) analysis (114). Strain *B. barranii* CC1502, which is the inoculant strain for *Chamaecytisus palmensis*, an introduced tree legume species, grouped with the introduced *Lotus pedunculatus* strain *B. barranii* CB829 (from the USA) on the core genome tree but clustered within the *Lupinus*/*Ornithopus* group within the symbiosis gene tree. This suggests that like WSM471 and WU425, CC1502 likely represents an exotic strain that has become naturalized to Australian soils.

For the remaining four inoculant strains isolated from Australia that nodulate tropical legumes, a complex picture of core and symbiosis gene phylogenies is evident. This is partly influenced by the paucity of host range data for many of these strains. Where host range data exist, there is a tendency for some *Bradyrhizobium* strains to fix N_2_ effectively across many legume genera. For example, *Bradyrhizobium* sp. CB756 is effective on species across 19 genera, *Bradyrhizobium* sp. CB1015 is effective across eight genera, and *B. yuanmingense* CB1024 is effective across 15 genera (85). Nevertheless, the geographical origins of inoculant strains can still be inferred from core and symbiosis gene phylogenies of these organisms. Inoculant strain *B. huanghuaihainense* CB3035 for *Cyamopsis tetragonoloba* (native to India, Pakistan, and Western Himalaya) was most similar to *B. huanghuaihainense* CGMCC1.10948^T^ isolated from *Glycine max* from China, and more distantly similar to CB1717 for *Macroptilium bracteatum* (native to South America), which was isolated from Brazil, on the core genome tree, but its symbiosis genes were more closely related to *Bradyrhizobium* sp. CB1650, which is the commercial inoculant strain for *Stylosanthes hamata* from Brazil. In contrast, the commercial inoculant strain for a range of *Stylosanthes* spp. (excluding *S. hamata*), *Bradyrhizobium* sp. CB82, which was isolated from Australia, was only distantly related to CB1650 on the core and symbiosis genome trees, instead being more closely related to *B. arachidis* SM32 from China, and *B. centrolobii* BR 10245^T^ and *B. neotropicale* BR 10247^T^ isolated from *Centrolobium paraense* in Brazil on both trees (Fig. 5). Similarly, *Aeschynomene americana* and *A. falcata* inoculant strain *Bradyrhizobium* sp. CB2312, which was isolated in Australia, is closest to *B. arachidis* strain CB756 (from Zimbabwe) on both core and symbiosis gene trees but not to other *Aeschynomene* sp.-nodulating strains, which form a distinct cluster in both phylogenies and were isolated from the North America, Africa, and Japan (115–118). Finally, *B. brasilense* 5G1B, which is the inoculant strain for *Vigna angularis* isolated from Australia, is most closely related to *B. pachyrizi* CB1923 from Brazil, the inoculant strain for *Centrosema pascuorum* and *C. pubescens* at the core genome level, but is symbiotically closer to *G. max* strains *B. elkanii* USDA76^T^ and USDA61.

There is a suite of well-characterized strains of *Bradyrhizobium* spp. isolated from Australian native legumes including *B. agreste* CNPSo 4010^T^, *B. archetypum* WSM1744^T^, *B. australiense* WSM1791^T^, *B. cenepequi* CNPSo 4026^T^, *B. commune* BDV 5040^T^, *B. diversitatis* CNPSo 4019^T^, *B. glycinis* CNPSo 4016^T^, *B. hereditatis* WSM1704^T^, and *B. murdochi* WSM1790^T^. The genome sequences of these strains do not cluster with the seven *Bradyrhizobium* sp. inoculants isolated from Australia on the core gene tree. This suggests that these seven inoculant strains are exotic organisms. However, we cannot rule out the possibility that they represent geographically widespread organisms that could also have broad host ranges.

### Conclusions and future perspectives

Australian commercial legume inoculants are composed of a wide diversity of organisms, which span five known rhizobial genera and at least 19 different species. Twenty-three strains could be definitively identified at the species level, while 19 strains could only be conclusively defined at the genus level. With our knowledge of rhizobia-legume symbioses built upon a narrow suite of strains and host organisms (1, 119, 120), sequencing of a greater diversity of rhizobia is required to bolster databases and make them more representative of rhizobial populations.

Within the genera analyzed in this study, varying degrees of incongruency between core and symbiosis gene phylogenies were observed, with the level of discordance suggesting a genus-based hierarchy of HGT frequency of *Mesorhizobium* > *Rhizobium* > *Sinorhizobium* > *Bradyrhizobium*. While there is clear evidence for HGT and its impact on N_2_ fixation for *Mesorhizobium* spp. in the field (9, 10, 77, 121), the same is not true for the other rhizobia genera. For *Rhizobium* spp. and *Sinorhizobium* spp., where pSyms have been shown to be mobile *in vitro* (14, 15), their environmental transfer is yet to be directly observed. Similarly, several studies have concluded symbiosis gene HGT is likely an important driver for the evolution of *Bradyrhizobium* spp. symbionts (58, 122–124), but the mechanism of transfer for these genes has yet to be elucidated.

Inoculant strains isolated from Australian soils were shown to be similar to exotic strains. This suggests that these inoculants are the result of inadvertent introductions of rhizobia, possibly arriving along with exotic soil, legume seed, or plant material, colonizing Australian soils and subsequently being isolated and developed as inoculants. However, with a paucity of sequence data of native rhizobia, the possibility remains that some of these commercial strains may be indigenous bacteria with a capacity to fix N_2_ with introduced legumes. This is particularly pertinent for the *Bradyrhizobium* inoculant strains, where there is substantial evidence that native legumes are nodulated by this genus (125–129). Sequencing more rhizobia isolated from indigenous legume hosts would improve the resolution of this analysis and enable this question to be answered.

The rhizobia analyzed in this study are a cohort of strains with high saprophytic competence and N_2_ fixation efficiency for targeted host legumes. They provide a blueprint to allow the development of a sequence-based approach to identify nodule occupancy in the field. These genome sequences are also a highly valuable resource for interrogation into free-living persistence of rhizobia and their evolution as legume N_2_-fixing symbionts.

## Data Availability

The complete genome sequences were deposited to NCBI under BioProject accession number PRJNA783123.

## SUPPLEMENTAL MATERIAL

Supplemental tables aem.02213-24-s0001.docx Table S1, genomic location of symbiosis genes; Table S2, symbiosis islands detected in *Bradyrhizobium* spp.

ASM does not own the copyrights to Supplemental Material that may be linked to, or accessed through, an article. The authors have granted ASM a non-exclusive, world-wide license to publish the Supplemental Material files. Please contact the corresponding author directly for reuse.
