# Supplementary material for: Closed genomes of commercial inoculant rhizobia provide a blueprint for management of legume inoculation
Source: Appl Environ Microbiol. 2025 Jan 10;91(2):e02213-24. doi: 10.1128/aem.02213-24 (PMC11837538; doi:10.1128/aem.02213-24)
Supplement: Supplemental tables — Table S1, genomic location of symbiosis genes; Table S2, symbiosis islands detected in Bradyrhizobium spp. [file aem.02213-24-s0001.docx]

Table S1. Genomic location of common nodulation and nitrogen fixation genes within inoculant genomes.

| Strain | *nodABCIJ* | *nifHDKEN* | *fixABCX* | *fixNOQP* |
| --- | --- | --- | --- | --- |
| 5G1B | 6,946,214-6,952,975* | 7,506,285-7,505,401 (*nifH*), 7,524,724-7,531,023 (*nifDKEN*) | 6,923,529-6,922,687 (*fixA*), 7,498,704-7,501,465 (*fixBCX*) | 6,832,023-6,835,473 |
| CB82 | 7,591,422-7,598,141* | 7,545,780-7,553,016 | 7,621,478-7,620,618 (*fixA*), 7,524,577-7,527,344 (*fixBCX*) | 6,284,169-6,287,621^ |
| CB376 | 4,294,481-4,299,101 | 3,971,500-3,978,686 | 4,019,465-4,023,085 | 3,651,169-3,654,670 |
| CB627 | 7,238,647-7,245,410* | 7,784,468-7,783,584 (*nifH*), 7,801,666-7,807,968 (*nifDKEN*) | 7,230,357-7,229,515 (*fixA*), 7,776,900-7,779,667 (*fixBCX*) | 7,124,662-7,128,110 |
| CB756 | 2,270,023-2,276,783* | 1,695,854-1,696,738 (*nifH*), 1,669,456-1,675,741 (*nifDKEN*) | 1,915,159-1,915,908 (*fixA1*), 2,099,897-2,099,148 (*fixA2*), 2,284,559-2,285,401 (*fixA3*), 9,167,631-9,166,882 *(fixA4*)^, 1,700,719-1,703,483 (*fixBCX*) | 3,111,728-3,115,182^ |
| CB782 | 101,022-105,411 [pCB782_3] | 58,184-66,312 [pCB782_3]^#^ | 53,915-57,509 [pCB782_3] | 112,083-115,670 [pCB782_3] |
| CB1015 | 1,721,559-1,728,318* | 1,318,519-1,319,403 (*nifH*), 1,293,270-1,299,551 (*nifDKEN*) | 1,734,356-1,735,219 (*fixA1*), 7,989,932-7,989,183 (*fixA2*)^, 1,323,616-1,326,379 (*fixBCX*) | 3,155,604-3,159,060^ |
| CB1024 | 5,436,424-5,443,184* | 6,071,079-6,070,195 (*nifH*), 6,085,478-6,091,763 (*nifDKEN*) | 5,428,740-5,427,874 (*fixA1*), 6,858,183-6,857,434 (*fixA2*)^, 6,063,400-6,066,164 (*fixBCX*) | 2,011,185-2,014,641^ |
| CB1650 | 7,552,348-7,559,108* | 8,161,774-8,160,890 (*nifH*), 8,182,955-8,189,243 (*nifDKEN*) | 1,474,718-1,475,467 (*fixA1*)^, 7,544,438-7,543,596 (*fixA2*), 8,153,874-8,156,637 (*fixBCX*) | 6,390,667-6,394,119^ |
| CB1717 | 7,661,944-7,668,697* | 7,916,124-7,915,240 (*nifH*), 7,935,247-7,941,534 (*nifDKEN*) | 7,654,115-7,653,273 (*fixA1*), 8,581,154-8,580,405 (*fixA2*), 7,908,469-7,911,232 (*fixBCX*) | 7,070,703-7,074,156^ |
| CB1809 | 7,796,541-7,803,294* | 8,078,094-8,077,210 (*nifH*), 8,092,556-8,098,835 (*nifDKEN*) | 7,788,897-7,788,031 (*fixA1*), 8,509,476-8,508,727 (*fixA2*)^, 8,071,579-8,074,342 (*fixBCX*) | 2,954,354-2,957,807^ |
| CB1923 | 7,180,496-7,187,245* | 7,358,638-7,357,754 (*nifH*), 7,378,996-7,385,308 (*nifDKEN*) | 1,187,361-1,188,110 (*fixA1*)^, 7,173,299-7,172,457 (*fixA2*), 7,350,980-7,353,756 (*fixBCX*) | 6,995,083-6,998,532, 7,851,520-7,854,969^ |
| CB2312 | 8,389,013-8,395,776* | 8,706,015-8,705,131 (*nifH*), 8,722,844-8,729,129 (*nifDKEN*) | 1,097,480-1,098,229 (*fixA1*)^, 8,403,530-8,404,372 (*fixA2*), 8,698,369-8,701,133 (*fixBCX*) | 7,247,093-7,250,547^ |
| CB3035 | 7,978,542-7,985,302* | 8,661,009-8,660,125 (*nifH*), 8,677,654-8,683,939 (*nifDKEN*) | 1,655,191-1,655,940 (*fixA1*)^, 7,970,783-7,969,941 (*fixA2*), 8,337,372-8,336,623 (*fixA3*), 8,653,121-8,655,884 (*fixBCX*) | 6,827,906-6,831,359^ |
| CB3060 | 116,793-116,203 (*nodA1*) [pCB3060_2], 166,409-166,999 (*nodA2*) [pCB3060_2], 204,775-211,479 [pCB3060_2]* | 253,198-260,310 [pCB3060_2] | 218,673-220,391 (*fixAB*) [pCB3060_1], 264,018-265,787 (*fixAB*) [pCB3060_1], 227,497-231,102 [pCB3060_2], 101,768-103,425 (*fixAB*) [pCB3060_3], 203,804-205,610 (*fixAB*) [pCB3060_3] | 179,840-183,222 [pCB3060_2] |
| CB3090 | 121,834-122,424 (*nodA1*) [pCB3090_2], 173,124-173,714 (*nodA2*) [pCB3090_2], 214,138-220,843 [pCB3090_2] | 267,873-274,988 [pCB3090_2] | 101,427-103,133 (*fixAB*) [pCB3090_1], 243,230-245,206 [pCB3090_2], 1,580,761-1,582,463 (*fixAB*) [pCB3090_1], 2,956,542-2,958,248 (*fixAB*) [Chr] | 190,020-193,402 [pCB3090_2], 65,064-68,457 [pCB3090_3] |
| CB3126 | 493,653-500,357 [pCB3126_2]*, 509,940-510,530 (*nodA*) [pCB3126_2] | 371,899-379,022 [pCB3090_2] | 451,140-454,747 [pCB3126_2], 2,810,876-2,812,671 (fixAB) [Chr] | 757,580-761,059 [pCB3126_1], 1,751,573-1,755,050 [Chr] |
| CB3171 | 216,587-223,292 [pCB3171_2]*, 120,737-121,327 (*nodA*) [pCB3171_2], 170,708-171,298 (*nodA*) [pCB3171_2] | 3: 264,474-271,589 [pCB3171_2] | 221,469-223,181 (*fixAB*) [pCB3171_1], 266,813-268,531 (*fixAB*) [pCB3171_1], 2,451,893-2,453,656 (*fixAB*) [pCB3171_1], 239,593-243,196 [pCB3171_2], 2,982,784-2,984,502 (*fixAB*) [Chr] | 2,271,038-2,274,424 [pCB3171_1], 190,343-193,725 [pCB3171_2] |
| CB3481 | 5,202,128-5,208,882* | 5,550,985-5,551,869 (*nifH*), 5,578,380-5,584,697 (*nifDKEN*) | 963,539-964,288 (*fixA1*)^, 5,169,289-5,170,131 (*fixA2*), 5,544,451-5,547,222 (*fixBCX*) | 4,192,025-4,195,448^, 5,686,386-5,689,804 |
| CC283b | 395,604-399,994 [pCC283b_4] | 408,271-415,268 [pCC283b_4] | 832,026-833,730 [pCC283b_1], 403,984-407,584 [pCC283b_4], 4,157,165-4,158,869 [Chr] | 458,192-461,583 (pCC283b_4], 165,529-168,986 [Chr] |
| CC511 | 360,468-361,055 (*nodA*) [pCC511_4], 328,167-332,858 (*nodBCSIJ*) [pCC511_4] | 251,662-258,744 [pCC511_4], 312,920-313,813 (*nifH*) [pCC511_4], 354,007-358,174 (*nifHDK*) [pCC511_4] | 457,026-458,730 (*fixAB*) [pCC511_2], 269,933-273,535 [pCC511_4], 3,756,083-3,757,789 (*fixAB*) [Chr] | 343,103-346,494 [pCC511_4] |
| CC829 | 1,514,509-1,521,261 [Chr]* | 1,364,769-1,365,659 (*nifH*) [Chr], 1,342,846-1,349,142 (*nifDKEN*) [Chr] | 1,528,780-1,529,646 (*fixA1*) [Chr], 9,017,172-9,016,423 (*fixA2*) [Chr]^, 1,369,809-1,372,577 (*fixBCX*) [Chr] | 2,556,556-2,560,010 [Chr]^ |
| CC1192 | 4,574,355-4,578,910 [Chr] | 4,517,451-4,524,512 [Chr] | 4,551,698-4,555,302 [Chr] | 4,228,864-4,232,241 [Chr] |
| CC1502 | 1,979,553-1,986,300* [Chr] | 1,600,275-1,601,165 (*nifH*) [Chr], 1,476,872-1,483,295 (*nifDKEN*) [Chr] | 1,863,546-1,864,295 (*fixA1*) [Chr], 1,997,339-1,998,205 (*fixA2*) [Chr], 9,107,268-9,108,017 (*fixA3*) [Chr]^, 1,605,089-1,607,857 (*fixBCX*) [Chr] | 2,901,213-2,904,667 [Chr]^ |
| CIAT3101 | 8,389,238-8,395,929* | 8,345,628-8,352,866 | 1,028,938-1,029,687 (*fixA1*)^, 8,417,825-8,418,685 (*fixA2*), 8,325,552-8,328,317 (*fixBCX*) | 6,646,329-6,649,783^ |
| NC92 | 6,861,557-6,868,309* | 7,388,141-7,389,025 (*nifH*), 7,402,668-7,408,954 (*nifDKEN*) | 6,849,469-6,850,308 (*fixA1*), 7,874,373-7,875,122 (*fixA2*), 7,381,121-7,383,881 (*fixBCX*) | 2,517,068-2,520,524^ |
| RRI128 | 608,865-613,267 [pRRI128_2] | 582,525-588,133 (*nifHDKE*) [pRRI128_2], 615,480-616,805 (*nifN*) [pRRI128_2] | 576,709-580,317 [pRRI128_2], 814,191-815,986 (*fixAB*) [pRRI128_2], 2,565,252-2,566,954 (*fixAB*) [pRRI128_1] | 458,414-461,890 [pRRI128_2], 548,366-551,761 [pRRI128_2] 715,038-718,440 [pRRI128_2] |
| SRDI969 | 3,926,615-3,931,007 [Chr] | 3,951,943-3,958,952 [Chr] | 3,916,179-3,919,786 [Chr], 4,118,655-4,120,359 (fixAB) [Chr] | 3,209,789-3,213,182 [Chr], 3,908,381-3,911,772 [Chr] |
| SU277 | 763,460-767,862 [pSU277_2] | 794,935-800,543 (*nifHDKE*) [pSU277_2], 759,924-761,249 (*nifN*) [pSU277_2] | 802,747-806,355 [pSU277_2], 2,592,538-2,594,240 (*fixAB*) [Chr] | 380,700-384,102 [pSU277_2], 389,985-393,461 [pSU277_2], 837,976-841,378 [pSU277_2] |
| SU303 | 490,758-495,150 [pSU303_1] | 459,402-466,402 [pSU303_1] | 502,456-506,063 [pSU303_1], 669,661-671,365 (fixAB) [pSU303_1], 4,194,462-4,196,177 (*fixAB*) [pSU303_1] | 510,470-513,861 [pSU303_1], 809,147-812,604 [Chr] |
| SU343 | 6,395,296-6,395,955 (nodB) [Chr], 6,403,249-6,407,036 (nodACIJ) [Chr], 6,754,581-6,755,171 (nodA) [Chr] | 6,675,602-6,682,719 [Chr] | 1,634,142-1,635,844 (*fixAB*) [Chr], 6,718,289-6,721,898 [Chr] | 2,786,291-2,789,669 [Chr], 6,290,155-6,293,534 [Chr] |
| TA1 | 347,395-351,794 [pTA1_4] | 331,642-338,608 [pTA1_4] | 339,174-342,778 [pTA1_4], 442,310-444,012 (*fixAB*) [pTA1_1], 4,144,696-4,146,398 (*fixAB*) [Chr] | 180,371-183,827 [Chr], 4,735,554-4,738,945 [Chr] |
| WSM471 | 1,581,645-1,588,394* | 1,469,110-1,470,000 (*nifH*), 1,447,048-1,453,337 (*nifDKEN*) | 1,472,973-1,475,741 (*fixBCX*), 1,595,744-1,596,601 (*fixA1*), 1,882,554-1,883,303 (*fixA2*), 7,288,926-7,289,675 (*fixA3*) | 2,902,562-2,906,016^ |
| WSM1115 | 871,607-876,009 [pWSM1115_2] | 868,071-869,396 (*nifN*) [pWSM1115_2], 902,854-908,462 (*nifHDKE*) [pWSM1115_2] | 2,867,977-2,869,679 (*fixAB*) [Chr], 910,673-914,281 [pWSM1115_2] | 651,014-654,490 [pWSM1115_2], 660,373-663,775 [pWSM1115_2], 660,373-663,775 [pWSM1115_2] |
| WSM1274 | 690,731-695,123 [pWSM1274_1] | 663,004-669,974 [pWSM1274_1] | 637,772-639,601 (*fixAB*) [pWSM1274_1], 700,379-703,986 [pWSM1274_1], 940,273-941,977 (*fixAB*) [pWSM1274_1], 4,140,043-4,141,759 (*fixAB*) [Chr] | 708,328-711,719 [pWSM1274_1], 171,510-174,967 [Chr], 3,292,242-3,295,635 [Chr] |
| WSM1325 | 295,685-300,055 [pR132502] | 308,462-315,396 [pR132502] | 202,804-204,508 (*fixAB*) [pR132502], 304,306-307,910 [pR132502], 3,925,442-3,927,158 (*fixAB*) [Chr] | 340,868-344,259 [pR132502] |
| WSM1455 | 685,077-689,469 [pWSM1455_1] | 654,847-661,817 [pWSM1455_1] | 694,723-698,330 [pWSM1455_1], 809,302-811,018 (*fixAB*) [pWSM1455_1], 3,895,147-3,896,862 (*fixAB*) [Chr] | 699,215-702,606 [pWSM1455_1] |
| WSM1497 | 6,486,783-6,487,373 (nodA) [Chr], 6,492,103-6,496,627 [Chr] | 6,438,646-6,445,752 [Chr] | 1,639,080-1,640,917 (*fixAB*) [Chr], 6,463,950-6,467,557 [Chr] | 6,119,066-6,122,442 [Chr] |
| WSM1558 | 6,708,129-6,708,719 (*nodA*), 6,713,415-6,717,943 | 6,660,404-6,667,511 | 1,590,826-1,592,610 (*fixAB*)^, 1,971,356-1,973,098 (*fixAB*)^, 6,685,777-6,689,384 | 6,087,277-6,090,658, 6,291,592-6,294,968^ |
| WSM1592 | 206,067-208,032 (*nodBC*) [pWSM1592_2], 210,342-210,932 (*nodA*) [pWSM1592_2], 272,325-276,722 [pWSM1592_2] | 279,607-286,668 [pWSM1592_2] | 166,842-170,450 [pWSM1592_2], 3,215,483-3,217,185 [Chr] | 1,156,602-1,159,987 [pWSM1592_1], 2,170,552-2,174,006 [pWSM1592_1] |
| WSM4643 | 283,746-288,138 [pWSM4643_2] | 308,895-315,895 [pWSM4643_2] | 274,883-278,490 [pWSM4643_2], 399,294-401,010 (*fixAB*) [pWSM4643_2], 4,037,426-4,039,142 (*fixAB*) [pWSM4643] | 267,092-270,483 [pWSM4643_2], 360,720-364,111 [pWSM4643_2], 3,244,708-3,248,101 [Chr] |
| WU425 | 6,740,069-6,746,818* | 7,043,560-7,044,450 (*nifH*), 7,068,812-7,075,102 (*nifDKEN*) | 1,183,270-1,184,019 (*fixA1*)^, 6,731,776-6,732,633 (*fixA2*), 6,832,827-6,833,576 (*fixA3*), 7,037,829-7,040,597 (fixBCX) | 5,880,048-5,883,502^ |

*includes nodSU, ^not on SI, ^#^nifD and nifK separated by 3 thioredoxin CDS; parenthesis ( ) denote gene names in instances in which the operon is separated into multiple parts, brackets [ ] denote the replicon containing operon in multipartite genomes.

Table S2. Symbiosis islands detected in *Bradyrhizobium* spp. inoculants.

| Strain | A | | B | | C | | tRNA | *att* site |
| --- | --- | --- | --- | --- | --- | --- | --- | --- |
|  | Size | Position | Size | Position | Size | Position |  |  |
| 5G1B | 1,186,148 | 6,476,046 -7,662,193 | - | - | - | - | tRNA-Glu | - |
| CB82 | 1,005,155 | 7,418,042-8,423,196 | 246,631 | 1,079,418-1,326,048 | - | - | tRNA-Val | TTCACACGGGAGAGGTCCAAGGTTCGATCCCTTGTGCGCCCACCA |
| CB627 | 823,972 | 7,038,267-7,862,238 | - | - | - | - | tRNA-Glu | - |
| CB756 | 881,791 | 1,615,208-2,496,998 | - | - | - | - | tRNA-Ile | - |
| CB1015 | 577,690 | 1,223,711-1,801,400 | 194,289 | 1,807,983-2,002,271 | - | - | tRNA-Val | CCTTGACATGGTAGGGGTCACAGGTTCGATCCCTGTCGTGCCCACCATCCT |
| CB1024 | 845,148 | 5,292,435-6,137,582 | - | - | - | - | tRNA-Ile | - |
| CB1650 | 1,098,987 | 7,401,457-8,500,443 | - | - | - | - | tRNA-Val | - |
| CB1717 | 647,706 | 7,348,046-7,995,751 | - | - | - | - | tRNA-Ile | - |
| CB1809 | 672,335 | 7,647,515-8,319,849 | 15,849 | 1,881,954-1,897,802 | 156,256 | 841,128-997,383 | tRNA-Val | TTCACACGGGAGAGGTCCAAGGTTCGATCCCTTGTGCGCCCACCAT |
| CB1923 | 636,927 | 6,965,608-7,602,534 | - | - | - | - | tRNA-Val | TTCACACGGGAGAGGTCCAAGGTTCGATCCCTTGTGCGTCCACCATTAA |
| CB2312 | 899,892 | 7,881,484-8,781,375 | - | - | - | - | tRNA-Ile | - |
| CB3035 | 1,208,964 | 7,601,657-8,810,620 | - | - | - | - | tRNA-Val | - |
| CB3481 | 772,783 | 5,109,476-5,882,258 | - | - | - | - | - | - |
| CC829 | 485,737 | 1,297,990-1,783,726 | 37,561 | 8,417,616-8,455,176 | - | - | tRNA-Val | TTCACACGGGAGAGGTCCAAGGTTCGATCCCTTGTGCGCCCACCAT |
| CC1502 | 761,246 | 1,337,856-2,099,101 | 167,633 | 8,345,494-8,513,126 | 362,520 | 8,345,494-8,513,126 | tRNA-Val | TTCACACGGGAGAGGTCCAAGGTTCGATCCCTTGTGCGCCCACCATT |
| CIAT3101 | 605,622 | 8,213,550-8,819,171 | 858,788 | 1,850,582-2,709,369 | - | - | tRNA-Lys | GCAGCTGACTCTTAATCAGCGGGTCCCAGGTTCGAGCCCTGGTGCGCCCACCA |
| NC92 | 844,827 | 6,670,456-7,515,282 | 464,902 | 1,348,709-1,813,610 | - | - | tRNA-Ile | ACGGTCTGGTTGCAGGTTCGAGTCCTGCCGGGCCCACCAATAAA |
| WSM471 | 569,250 | 1,401,860-1,971,109 | 43,778 | 6,720,178-6,763,955 | - | - | tRNA-Val | ATGGTGGGCGCACAAGGGATCGAACCTTGGACCTCTCCCGTGTGAA |
| WU425 | 569,581 | 6,556,378-7,125,958 | 55,508 | 1,698,508-1,754,015 | - | - | tRNA-Val | TTCACACGGGAGAGGTCCAAGGTTCGATCCCTTGTGCGCCCACCATT |
